# On the Question of the Full Selective Synthesis of Potentially Bioactive of 2-(*tert*-Butyl)-3-hydroxy-7-2,3-dihydro-1*H*-pyrrolo[3,4-c]pyridin-1-ones and Their Derivatives: Experimental and DFT Computational Study

**DOI:** 10.3390/molecules31111973

**Published:** 2026-06-05

**Authors:** Magdalena Ciechańska, Ewelina Wielgus, Rafał Dolot, Andrzej Jóźwiak, Radomir Jasiński

**Affiliations:** 1 Department of Organic Chemistry, Faculty of Chemistry, University of Lodz, Tamka 12, 91-403 Łódź, Poland; 2 Centre of Molecular and Macromolecular Studies, Polish Academy of Sciences, Sienkiewicza 112, 90-363 Łódź, Poland; ewelina.wielgus@cbmm.lodz.pl (E.W.); rafal.dolot@cbmm.lodz.pl (R.D.); 3 Cracow University of Technology, Department of Organic Chemistry and Technology, Warszawska 24, 31-155 Kraków, Poland

**Keywords:** pyrrolo[3,4-c]pyridine, direct lithiation, DFT calculations

## Abstract

The practical aspects of the full regioselective preparation of 2-(*tert*-butyl)-3-hydroxy-7-2,3-dihydro-1*H*-pyrrolo[3,4-c]pyridine-1-ones and their derivatives were described. Created in our laboratory, the reaction protocol is simple and occurs under mild conditions. It is important that all obtained products are stable, pure, crystalline and can be easily identified based on spectral data and X-ray analysis results. Key aspects of the reaction course were explained based on the DFT quantum chemical calculations.

## 1. Introduction

The pharmacological properties of pyrrolo[3,4-*c*]pyridine derivatives are one of the main reasons for the development of new compounds containing this core [1,2,3,4,5,6]. The building block of 3-hydroxy-2,3-dihydro-1*H*-pyrrolo[3,4-*c*]pyridin-1-one **1** is present in substance **2** belonging to the class of antituberculous compounds that inhibit the cytochrome bc1 complex [7]. Fluorine derivative **3** was used in the synthesis iso-citrate dehydrogenase inhibitor [8]. Substances containing the core **1** are also useful intermediates for inter alia, the manufacturing of compounds with antiproliferative activity [9], anti-infectious derivatives [10], as well as for organic electronic elements [11]. Substituted 2,3-dihydro-1*H*-pyrrolo[3,4-c]pyridines constitute an important class of fused heterocyclic compounds due to their presence in numerous biologically active molecules. These derivatives exhibit diverse pharmacological activities. Some pyrrolo[3,4-c]pyridine compounds have been reported to have potential analgesic and sedative activity [12,13]. Derivatives bearing a carboxyl group are known to inhibit aldose reductase activity, which is relevant in the treatment of diabetic complications [14]. Furthermore, 7-hydroxy-1,3-dioxo-2,3-dihydro-1*H*-pyrrolo[3,4-c]pyridine-4-carboxylates have demonstrated HIV-1 inhibitory properties [15]. In addition, urea-based derivatives of this heterocyclic system act as potent inhibitors of human nicotinamide phosphoribosyltransferase (NAMPT), an enzyme involved in NAD biosynthesis [16]. To discover new NAMPT inhibitors with enhanced activity, improved toxicological profiles, and favorable physicochemical properties, bioisosteric replacements for the cyanoguanidine moiety present in NAMPT inhibitor structures were investigated. For this purpose, pyrrolo[3,4-c]pyridine derivatives **4** and **5** containing a sulfonamide group moiety linked to the pyrrolo nitrogen atom by a flexible aliphatic linker were synthesized [17].

An additional interesting aspect are chemosensors based on the core of pyrrolo[3,4-c]pyridine. This probe demonstrates excellent fluorescence selectivity for Fe^3+^ and Fe^2+^ ions compared to other common metal cations. Furthermore, in vitro fluorescence imaging studies reveal that its biocompatibility and low cytotoxicity make it highly suitable for detecting Fe^3+^ ions in biological samples [18].

Similarly, 1*H*-pyrrolo[3,4-*c*]pyridine-1,3(2*H*)-diones have been described as drugs, and bioactive compounds and the substance showed aggregation-induced emission characteristics [19]. Moreover, pyrrolo[3,4-c]pyridine-1,3(2*H*)-dione derivatives have been used for the synthesis of the azaphenanthrene alkaloid Eupolauramine **6**, which exhibits potential biological properties. The use of these derivatives as precursors is strategic because they provide a rigid bicyclic scaffold that facilitates ring assembly. Their functional groups allow for selective transformations, leading to complex polycyclic systems [4].

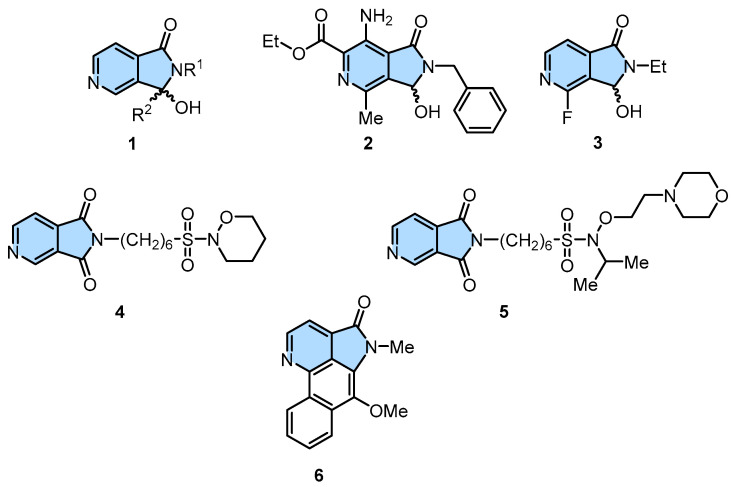



Directed lithiation has been employed for a variety of derivatives and has emergend as an effective strategy for modifying ring systems [20,21,22,23,24,25,26,27]. The action of organolithium compounds or lithium amides on 2,3-dihydroisoindol-1-ones (phtalimidinones) may lead to the formation of isoindoles [28,29,30,31,32], lithiation at position 3 [33,34,35,36,37,38], as well as to the lithiation of isoindolinone species at position 7 [39,40,41,42]. Herein, we wish to report a useful method for the functionalization of core **1** at seventh-position, selecting the position 2 *tert*-butyl group as it promotes the *ortho*-metallation process [43]. The course of key stages of the transformations described was explained and interpreted on the basis of DFT quantum chemical calculations.

## 2. Results

**Scheme 1:**
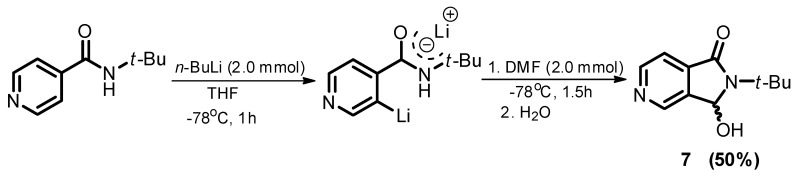
The general scheme of the synthesis of hydroxylactam **7**.

Our study started from the preparation of hydroxylactam **7**. We decided to prepare this compound by the lithiation of *N*-(*tert*-butyl)isonicotinamide using *n*-BuLi in THF, followed by the reaction of the resultant bislithiated amide with DMF (Scheme 1). The reaction product can be isolated by simple crystallization from the hexane:ethyl acetate system.

Its constitution was confirmed using elemental analysis data and spectral characteristics (see experimental part and the Supplementary Material). The IR spectrum displays a characteristic signal originating from the carbonyl group. In the ^1^H NMR spectrum, key diagnostic signals originating from all hydrogen atoms can be identified. Thus, in the weakest field (7.57–8.81 ppm), signals from the pyridine ring protons are present. In the moderate field, signals from the OH group proton and the conjugating proton associated with the five-membered ring are present. Finally, in the stronger field (1.53 ppm), a large singlet derived from the non-coupled protons of the *tert*-butyl group is present.

**Figure 1:**
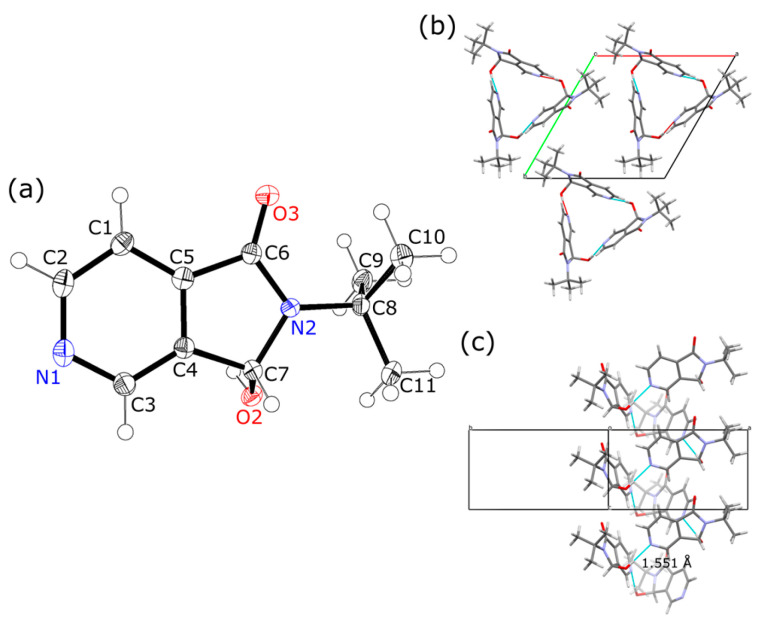
(**a**) Thermal ellipsoid representation of the asymmetric unit for the trigonal (*P*3_1_, Z′ = 1) form of compound **7**. The anisotropic displacement parameters (ADPs) are shown at the 50% probability level. (**b**) View of molecular packing along the crystallographic *c*-axis. (**c**) View along the diagonal a × b plane, showing the helical arrangement of the molecules with indications of intermolecular hydrogen contacts.

The characterization of compound **7** is complemented by ^1^H NMR, ^13^C NMR and MS spectra. Finally, its isomerism is further confirmed by X-ray structural analysis, showing that compound **7** crystallizes in the trigonal *P*3_1_ space group with one molecule per asymmetric unit. The crystal structure unequivocally confirmed that the crystallized molecule is the R-enantiomer at the C7 carbon atom (Figure 1a). Analysis of the molecular packing in the crystal shows that this position is stabilized by the O2–H2⋯N1(i) hydrogen bond (symmetry code: (i) 1+y−x, 1−x, −1/3+z) in of 1.991 Å, leading to the formation of infinite columns with a triangular cross-section (Figure 1b), arranged parallel to the *c*-axis, in which the hydroxylactam **7** molecules are arranged helically (Figure 1c). The space between the columns allows free movement of the solvent (methanol), whose position is not stabilized and was refined using a solvent mask, with 0.66 methanol molecule per one molecule of hydroxylactam **7**.

Compound **7** in the reaction in THF with 2.0 mole equivalents of *n*-BuLi in the presence of TMEDA was efficiently converted into the bis-(O^3−^ and C^7−^)-lithiated species **8**. Treatment of the lithiated compound with electrophiles after hydrolysis workup afforded, in all cases, the corresponding which was substituted at the seventh-position product number **9** and additionally bis-substituted -(O^3−^ and C^7−^) product **10**. The yields of products **9** and the recovered hydroxylactam **7** are summarized in Table 1. All obtained products were full characterized by respective physical data. The characterization of compound **9c** is complemented by ^1^H NMR, ^13^C NMR and HRMS spectra. The positive ESI mass spectrum reveals that compound **9c** is characterized by a protonated molecular ion and several fragment ions. Compound **9c** exhibits a protonated molecular ion at *m*/*z* 326.1514 ([M + H]^+^), consistent with the molecular formula C_18_H_19_N_3_O_3_. The fragmentation pattern shows neutral losses of H_2_O (*m*/*z* 308.1399) and C_4_H_8_ (*m*/*z* 270.0879). Ions at *m*/*z* 177.0300 and 151.0508 arise from fragmentation pathways involving the *tert*-butyl group and the phenyl-containing amide substituent (Ph–NH–CO–), respectively.

**Figure 2:**
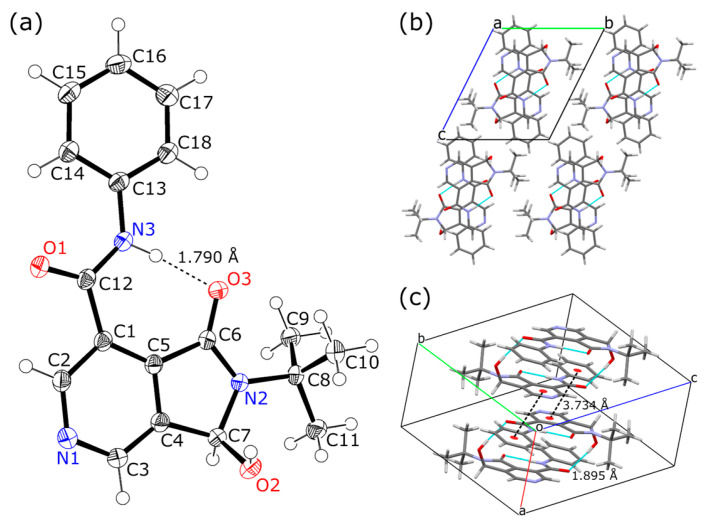
(**a**) Thermal ellipsoid representation of the asymmetric unit for the triclinic (*P*-1, Z′ = 1) form of compound **9c**. The anisotropic displacement parameters (ADPs) are shown at the 50% probability level. (**b**) View of molecular packing along the crystallographic *a*-axis shows hydrophobic interactions between side chains. (**c**) Perspective view of molecular packing with the indication of π–π stacking interactions and intermolecular hydrogen bonds.

Finally, the isomerism of compound **9c** was confirmed by X-ray structural analysis. Compound **9c** crystallizes in the triclinic *P*-1 space group with one molecule per asymmetric unit. The crystal structure confirmed that the crystallized molecule is the S-enantiomer at the C7 carbon atom (Figure 2a). Only the S-enantiomer was successfully crystallized. The R-enantiomer could not be obtained in crystalline form. The molecular conformation is stabilized by an intramolecular N3–H3⋯O3 hydrogen bond measuring 1.790 Å, as well as intermolecular O2–H2···O1(i) hydrogen bond (symmetry code: (i) 2−x, 1−y, 1−z) between alternating molecules in the range of 1.895 Å. Dimers form stacked columns parallel to the crystal *a*-axis, with π–π stacking interactions between phenyl and pyridine rings, and with distances ranging from 3.734 Å (Figure 2c). Adjacent columns interact through hydrophobic interactions involving *tert*-butyl groups (Figure 2b). No solvent is present in the crystal structure.

**Figure 3:**
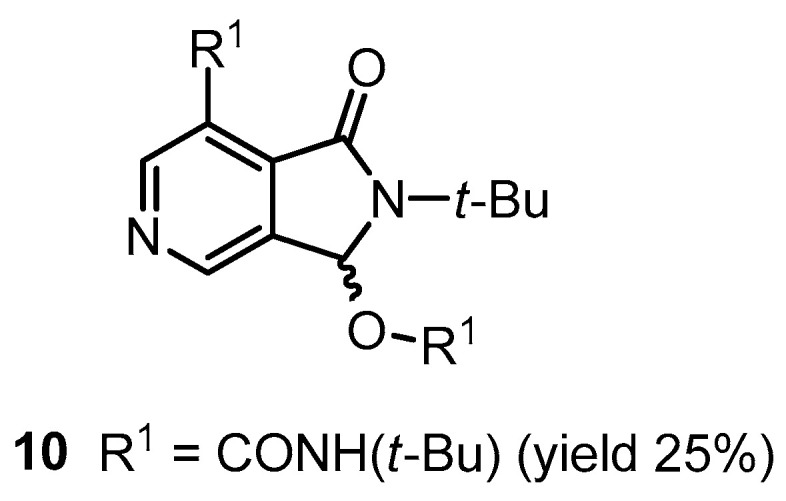
The molecular structure of the product **10**.

All products received were identified in a similar manner (see experimental part and the Supplementary Material). The deuteriation experiment showed quantitative conversion **7** into **9a**. Formation of bis-(O^3−^ and C^7−^) product **10** (Figure 3) was also observed when 2-isocyanato-2-methylpropane is used as an electrophile. The observed efficiencies indicate that in the tested processes, the reactivity of the electrophiles used is varied and determines the final result. The reaction conditions were optimized by varying the amounts of *n*-butyllithium, TMEDA and the electrophile. Our studies revealed that the best results were obtained when using *n*-BuLi (2 mmol) in the presence of TMEDA (2 mmol), whereas higher excess (2.5 and 3 mmol) of *n*-BuLi and TMEDA did not provide further improvement. The presence of TMEDA proved crucial, most likely due to its ability to enhance the reactivity and solvation of the organolithium species. In its absence, the reaction proceeded with noticeably lower efficiency and reproducibility. The optimal conditions involved lithiation at −78 °C for 1 h, followed by the addition of the electrophile (2 mmol) at the same temperature. Shorter times resulted in incomplete deprotonation, while extended times did not significantly improve yields. Increasing the excess of the electrophile (2.5 and 3 mmol) in these cases (entries 3, 4 and 5) led to the formation of mixtures of unidentified products, while the product was obtained in lower yield. This is most likely related to the high reactivity of such systems. Lithiation was most efficient at −78 °C (dry ice/acetone bath). At higher temperatures (−40 °C or 0 °C), an increased formation of by-products was observed, presumably due to competing side reactions. The progress of the reaction and the consumption of starting materials were monitored by thin-layer chromatography (TLC). TLC analysis allowed the continuous evaluation of substrate conversion as well as the detection of by-product formation. Based on TLC observations, the reaction time was not the same for all examples and depended on the nature of the electrophile and the reaction conditions. In some cases, the reactions proceeded more rapidly and afforded cleaner products, whereas, in others, prolonged reaction times promoted the formation of by-product mixtures. These results indicate the high reactivity of the investigated systems and their sensitivity to variations in reaction conditions.

**Scheme 2:**
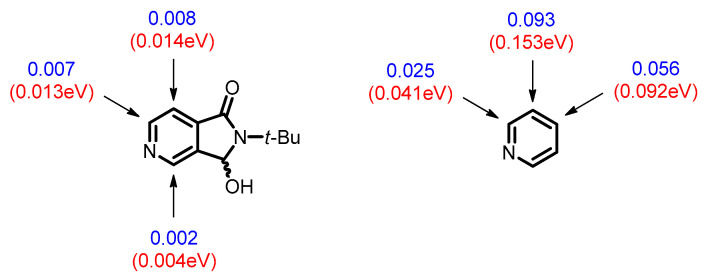
Local distribution of Parr spin densities (in blue) and nucleophilicity (in red) within molecule **7** and within the pyridine molecule.

Regardless of the reaction system used, the functionalization of the starting molecule **7** always occurs at the same position. This phenomenon can be explained based on the results of ωB97xD/6-311+G(d,p) (PCM) quantum chemical calculations. Recently, a similar approach was successfully used to explain and interpret the regiochemistry of many different polar bimolecular processes [44,45,46,47,48]. It was found that the value of the global nucleophilicity index (1.8 eV) for structure **7** allows (within the universal reactivity scale [49,50]) the classification of the molecule as a strong nucleophile. Interestingly, however, the local nucleophilicities on the pyridine segment atoms are relatively small and smaller than in pyridine (Scheme 2). However, the quantitative distribution of nucleophilicity is analogous in these molecules. In particular, the meta position in the pyridine segment is clearly the more active reaction center. Therefore, the actual lithiation process of molecule **7** is fully consistent with the nature of electrophile–nucleophile interactions in an elementary reaction.

It is interesting that thermodynamic factors, additionally, strongly favor the formation of product **8′**. In light of the data summarized in Table 2, the Gibbs free energies of the formation of isomeric lithiated lactams **8″** and **8″′** are more than 7 kcal/mol higher than in the case of compound **8**. This means that from a thermodynamic point of view, their formation is unlikely. It should be mentioned that in aromatic molecules with fused rings, each non-equivalent C-H bond has different acidity. One could suggest that the regioselectivity of the reaction is solely due to the difference in acidity of the non-equivalent CH bonds and that the product distribution is thermodynamically controlled. Also, lithium cation typically forms multi-coordinated complexes, which can direct the reaction toward a product that would not be formed with a non-lithium base.

**Figure 4:**
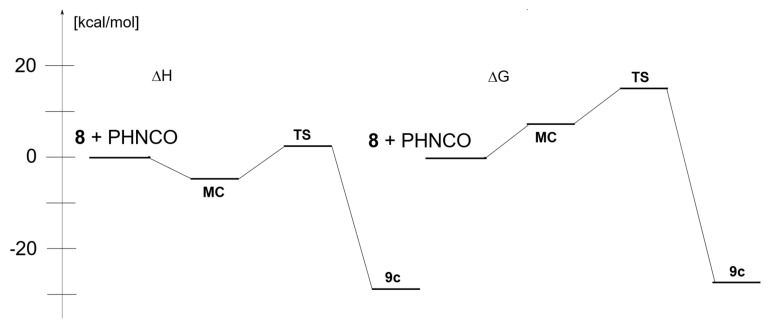
Energy profiles for the model process of introducing the electrophilic segment into the lithiated lactam **8** according to the ωB97xD/6-311+G(d,p) (PCM) calculations.

**Figure 5:**
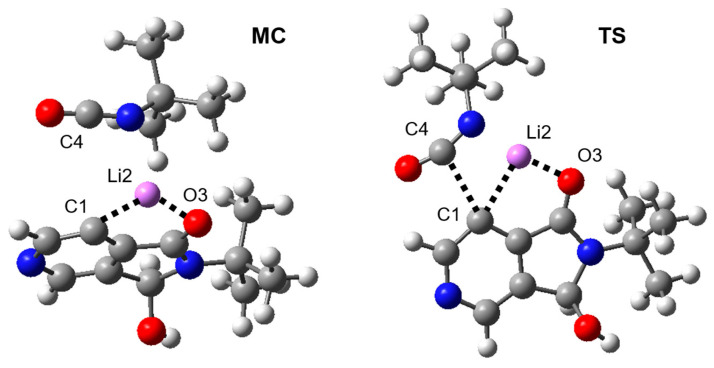
Views of critical structures for the model process of introducing the electrophilic segment into the lithiated lactam **8** according to the wB97xD/6-311+G(d,p) (PCM) calculations.

Finally, we decided to diagnose the mechanism of the key stage of introducing the electrophilic segment into the lithiated lactam, as it is very likely that this is not a typical, generally known substitution process [51,52,53]. We investigated this mechanism using the model illustrated in Figure 4 and Figure 5. It turned out that the crucial step of introducing the electrophilic segment onto the carbon atom of the five-membered ring in place of the lithium (C1 atom) should be treated as an addition process. In the first stage of this transformation, a pre-reaction MC complex is formed. This process occurs less with a barrier and is associated with a reduction in the enthalpy of the reacting system by 5.51 kcal/mol. Importantly, no new chemical bonds are formed at this stage, although the reaction centers adopts the orientation observed later in the transition state. Bimolecular pre-reaction complexes characterized by the similar nature were recently detected in relation to various types of polar processes [54,55,56]. The further movement into the transition state region requires an external energy input. The activation enthalpy, however, is very low, at only 1.78 kcal/mol. Although the entropic factor corrects it to higher values, it is still a kinetically easy process and completely permissible at room temperature. It should be underlined that energy profiles shown in Figure 4 show that the reaction proceeds with a low barrier, being highly exothermic and exergonic. That implies that there is thermodynamic control of the reaction rather than kinetic. Within the TS, dissociation of the C1-Li2 bond occurs, with simultaneous transfer of lithium toward the O3 oxygen atom. Simultaneously, a new C1–C4 bond forms. This creates an adduct still containing a lithium atom, which is only then transformed into the target molecule during hydrolysis under experimental conditions.

**Scheme 3:**
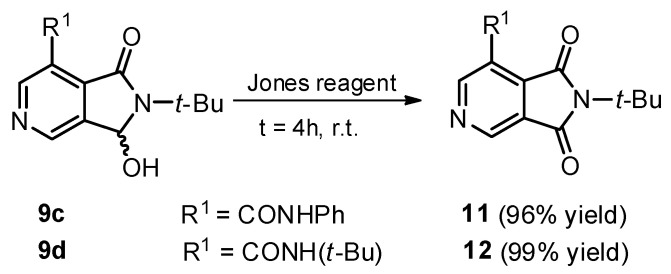
Synthesis of imides **11** and **12**.

Finally, we found that hydroxylactams (**9c**, **9d**) can be practically quantitatively converted, respectively, to imides (**11**, **12**) using Jones reagent (Scheme 3) [57]. As before, we identified the isolated imides spectrally (see experimental part and the Supplementary Material).

## 3. Materials and Methods

All reagents and commercially available materials were used without additional purification unless otherwise stated. *n*-Butyllithium (2.5 M solution in hexane) were purchased from Aldrich (St. Louis, MO, USA) and were analyzed immediately before use by applying the double titration procedure [58] with 1,2-dibromoethane in heptane solution (second step). *N*,*N*,*N*,*N*-Tetramethyl-1,2-ethylenediamine (TMEDA; Aldrich, 99.5%) was distilled prior to use and stored over KOH pellets. Chlorotrimethylsilane (purity 99%) was obtained from Fluka (Morris Plains, NJ, USA). Tetrahydrofuran (POCH, pure) was distilled from sodium benzophenone ketyl prior to use. Reagents and solvents were handled by using standard syringe techniques. All of the air- and moisture-sensitive reactions were carried out under an argon atmosphere. Analytical thin-layer chromatography (TLC) was conducted on Merck silica gel plates (Kieselgel 60 F254 (Merck Sp. Z o.o. Warszawa Poland), layer thickness 0.2 mm) with UV detection at 254 and/or 365 mm. Gravitational column chromatography (GCC) separations and purifications were performed on silica gel (0.063–0.100 mm) from Merck (Warszawa, Poland). ^1^H and ^13^C{1H} NMR spectra were obtained at ~294 K for solutions in CDCl_3_ or DMSO-d_6_ with a Brucker Avance III spectrometer at a frequency of 600.3 MHz (^1^H) and 150.9 MHz (^13^C) (Billerica, MA, USA), respectively, by using standard pulse sequences. The ^1^H signal of residual CHCl_3_ or DMSO-d_5_ and the ^13^C signal of the solvent were used as internal references (δH = 7.26 or 2.50 ppm and δC = 77.00 or 39.52 ppm, respectively) [59,60]. All NMR spectra were processed with the TopSpin 4.0.5 program; [61] the spectra are included in the SI. The splitting patterns are presented as follows: br = broad, m = multiplet, p = pseudo, s = singlet, d = doublet, t = triplet, q = quartet, dd = doublet of doublets, and so on. Infrared (IR) spectra were taken in KBr disks on a Thermo Nicolet Nexus FT-IR spectrometer (Thermo Fisher Scientific, Waltham, MA, USA); absorption frequencies are given in cm^−1^. Melting points (mp’s) were determined on a Boëtius microscope hot stage (Franz Küstner Nachfolger KG, Dresden, Germany) and are uncorrected. The analyses were carried out on a Vario EL III instrument (Elementar Analysensysteme GmbH, Langenselbold, Germany).

All high-resolution mass spectrometry (HRMS) measurements were performed using a Synapt G2-Si mass spectrometer (Waters, Milford, MA, USA) equipped with an electrospray ionization (ESI) source and a quadrupole time-of-flight mass analyzer. The mass spectrometer was operated in positive ion mode with the capillary voltage set to 3.0 kV and the sampling cone voltage set to 40 V. The source temperature was 100 °C. The scan range was *m*/*z* 50–1200, and the acquisition run time was 1 min.

To ensure accurate mass measurements, data were collected in centroid mode, and mass correction was applied during acquisition using a leucine encephalin solution as an external reference (LockSpray^TM^, Milford, MA, USA), which generated a reference ion at *m*/*z* 556.2771 ([M + H]^+^) in positive mode. The measurement data were processed using MassLynx 4.2 software (Waters) supplied with the instrument.

### 3.1. X-Ray Diffraction Analysis

X-ray quality crystals of compounds **7**, **9b**, **9c**, **9e**, **10**, **11** and **12** were formed over a period of approximately 3–5 days by re-crystallization from the solvent or the mixture of solvents, as described in Table S1. Suitable crystals were selected, transferred to mineral oil, and mounted on cryo loops. The crystals were then flash-cooled directly in a stream of N2. Diffraction intensities were recorded using a Rigaku XtaLAB Synergy-S diffractometer equipped with a Cu Kα radiation source (λ = 1.5418 Å) (Rigaku Americas Corporation, The Woodlands, TX, USA) and a HyPix-6000HE hybrid photon counting detector (Rigaku Tokyo, Japan). The total number of runs and images was based on the strategy calculation of the CrysAlisPro program (Rigaku, v 1.171.43.122a, 2024). The molecular models of the structures were created using the structure solution program SHELXT 2018/3, Olex2 1.5 [62] using intrinsic phasing with Olex2 (OlexSys Ltd., Durham, UK) [63] as the graphical interface and refined by least squares with the 2018/3 version of SHELXL [64]. All non-hydrogen atoms were refined anisotropically. The position of the hydrogen atoms was calculated geometrically and refined using the riding model. The structures were validated with CheckCif (http://checkcif.iucr.org accessed on 23 April 2026) and deposited in the Cambridge Crystallographic Data Center (CCDC) under the accession numbers 2548707, 2548713, 2548717, 2548718, 2548719, 254824 and 254828 for compounds **4**, **6b**, **6c**, **6e**, **8**, **9** and **10**, respectively. Images were prepared using the ORTEP-3 for Windows Ver.2020.1 [65] and Mercury 2025.3.0 (Build 466532) [66] software.

### 3.2. Preparation of N-(tert-butyl)isonicotinamide

*N*-(*tert*-butyl)isonicotinamide was obtained according to a known procedure [67] and was purified by crystallization from ethyl acetate/hexane (1:1) (yield 54%) m.p. 121–123 °C (ref. [68] m.p. 117–118 °C).

#### 3.2.1. Preparation of 2-(*tert*-Butyl)-3-hydroxy-2,3-dihydro-1*H*-pyrrolo[3,4-c]pyridine-1-one (**7**)

*N*-(*tert*-butyl)isonicotinamide (20.0 mmol) stirred in THF (80 mL) at −78 °C under argon was added BuLi (40.0 mmol). The solution was held at −78 °C for 1.0 h, and allowed to warm to 0 °C. The whole lot was cooled to −78 °C, and DMF (40.0 mmol) was added. The reaction mixture after 1.5 h at −78 °C was warmed to room temperature, kept for 1 h, and then added with water. The mixture was adjusted to pH ~7 with hydrochloric acid, and the organic layer was separated. The water layer was extracted with CHCl_3_. The combined organic solution was dried with magnesium sulfate(VI) and evaporated to give the crude products. The product was purified by crystallization from the mixture hexane:ethyl acetate 1:1.

2-(*tert*-butyl)-3-hydroxy-2,3-dihydro-1*H*-pyrrolo[3,4-c]pyridine-1-one (**7**).

White powder (2.552 g, 50% yield), R*_f_* = 0.18 (hexane:ethyl acetate 2:8), Mp: 152–154 °C. IR (KBr, cm^−1^): 1690 (C=O). ^1^H NMR (600 MHz, DMSO-d_6_): 8.81 (1H, s, 4-H), 8.74 (1H, d, *J* = 4.8 Hz, 6-H), 7.57 (1H, d, *J* = 4.8 Hz, 7-H), 6.55 (1H, d, *J* = 10.2 Hz OH), 6.21 (1H, d, *J* = 10.2 Hz, 3-H), 1.53 (9H, s, *t*-Bu). ^13^C{1 H} NMR (150 MHz, DMSO-d_6_): 165.7 (1), 150.8 (1), 145.4 (1), 140.4 (1), 139.7 (1), 116.6 (1), 81.5 (1), 54.6 (1), 28.3 (3). Anal. Calcd. for C_11_H_14_N_2_O_2_:C, 64.06; H, 6.84; N, 13.58; O, 15.52. Found C, 64.00: H, 6.92; N, 13.61. HRMS (+ESI): *m*/*z* calcd for C_11_H_14_N_2_O_2_ [M + H]^+^ 207.1134; found 207.1134.

#### 3.2.2. General Procedure for Transformation of Substrate **7** to Products **9**(**a**–**f**), **10**

To a stirred solution of 2-(*tert*-butyl)-3-hydroxy-2,3-dihydro-1*H*-pyrrolo[3,4-*c*]pyridin-1-one (4) (1 mmol) and TMEDA (2 mmol) in THF (30 mL), *n*-butyllithium (2.5 M in hexanes, 2 mmol) was added at −78 °C. The solution was kept at −78 °C for 1 h and an electrophile (2 mmol) was added. Stirring at −78 °C was continued for 1.5 h, and then the reaction mixture was warmed to r.t., followed by the addition of a saturated aqueous solution of ammonium chloride (5 mL). The products were extracted with dichloromethane, and the extracts were dried (Na_2_SO_4_) and evaporated to dryness. The crude products were purified by column chromatography (silica gel, methanol: petroleum ether 1:1 for **9b**, CH_2_Cl_2_: ethyl acetate 7:3) for **9a**, **9c**, **9d**, **9e**, **10** (hexane: ethyl acetate 3:7 for **9f**).

2-(*tert*-butyl)-3-hydroxy-2,3-dihydro-1*H*-pyrrolo[3,4-*c*]pyridin-1-one-7-*d* (**9a**).

White powder (204 mg, 98% yield, include 100% of deuterium at position 7). Mp: 153–154 °C. R*_f_* = 0.1 (CH_2_Cl_2_/ethyl acetate 7:3) IR (KBr, cm^−1^): 1689 (C=O). ^1^H NMR (600 MHz, DMSO-d_6_): 8.81 (1H, s, 4-H), 8.75 (1H, s, 6-H), 6.50 (1H, br. s, OH), 6.21 (1H, s, 3-H), 1.54 (9H, s, *t*-Bu). ^13^C{1 H} NMR (150 MHz, DMSO-*d*_6_): 165.1 (1), 150.0 (1), 144.7 (1), 140.0 (1), 139.3 (1), 116.0 (1), 81.0 (1), 54.1 (1), 27.8 (3). Anal. Calcd. for C_11_H_13_DN_2_O_2_:C, 63.75; H, 7.29; N, 13.52; O, 15.44. Found C, 63.80: H, 7.25; N, 13.56. HRMS (+ESI): *m*/*z* calcd for C_11_H_13_DN_2_O_2_ [M + H]^+^ 208.1196; found 208.1195.

2-(*tert*-butyl)-3-hydroxy-7-(trimethylsilyl)-2,3-dihydro-1*H*-pyrrolo[3,4-*c*]pyridin-1-one (**9b**). White powder (0.190 g, 70% yield). R*_f_* = 0.21 (methanol: petroleum ether 1:1). Mp: 190–192 °C. IR (KBr, cm^−1^): 1691 (C=O). ^1^H NMR (600 MHz, CDCl_3_): 8.53 (1H, s, 6-H), 8.41 (1H, s, 4-H), 6.09 (1H, s, 3-H), 4.83, (1H, br. s, OH), 1.62 (9H, s, *t*-Bu), 0.32 (9H, s, 7-SiMe_3_). ^13^C{1 H} NMR (150 MHz, CDCl_3_): 166.7 (1), 154.3 (1), 146.0 (1), 144.2 (1), 138.4 (1), 131.8 (1), 81.2 (1), 55.1 (1), 28.3 (3), −1.0 (3). Anal. Calcd. for C_14_H_22_N_2_O_2_Si: C, 60.39; H, 7.96; N, 10.06; Si, 10.09. Found C, 60.69: H, 7.75; N, 10.35. HRMS (+ESI): *m*/*z* calcd for C_14_H_22_N_2_O_2_Si [M + H]^+^ 279.1529; found 279.1530.

2-(*tert*-butyl)-3-hydroxy-1-oxo-*N*-phenyl-2,3-dihydro-1*H*-pyrrolo[3,4-*c*]pyridine-7-carboxamide (**9c**). White powder (0.180 g, 57% yield). R*_f_* = 0.88 (CH_2_Cl_2_: ethyl acetate 7:3), Mp: 246–247 °C. IR (KBr, cm^−1^): 1653, 1625 (C=O). ^1^H NMR (600 MHz, DMSO-d_6_): *δ* 12.63 (1H, s, N-H), 9.31 (1H, s, 4-H), 9.00 (1H, s, 6-H), 7.77 (2H, pd, *J* = 7.8 Hz, 2,6-Ph-H), 7.41 (2H, pt, *J* = 7.8 Hz, 3,5-Ph-H), 7.15 (1H, pt, *J* = 7.8 Hz, 4-Ph-H), 6.79 (1H, s, 3-H), 6.32 (1H, s, OH), 1.60 (9H, s, *t*-Bu-H). ^13^C{1 H} NMR (150 MHz, DMSO-*d*_6_): 166.8 (1), 161.4 (1), 152.0 (1), 148.0 (1), 139.9 (1), 139.1 (1), 136.6 (1), 129.5 (2), 125.6, (1) 124.5 (1), 120.1 (2), 81.3 (1), 55.7 (1), 28.0 (3). Anal. Calcd. for C_18_H_19_N_3_O_3_: C, 66.45; H, 5.89; N, 12.91. Found C, 66.39: H, 5.95; N, 12.86. HRMS (+ESI): *m*/*z* calcd for C_18_H_19_N_3_O_3_ [M + H]^+^ 326.1505; found 326.1514.

*N*,2-di-*tert*-butyl-3-hydroxy-1-oxo-2,3-dihydro-1*H*-pyrrolo[3,4-*c*]pyridine-7-carboxamide (**9d**). White powder (0.219 g, 73% yield). R*_f_* = 0.17 (CH_2_Cl_2_: ethyl acetate 7:3). Mp: 227–229 °C. IR (KBr, cm^−1^): 1672, 1639 (C=O). ^1^H NMR (600 MHz, CDCl_3_): 10.64 (1H, s, N-H), 9.09 (1H, s, 6-H), 8.51 (1H, s, 4-H), 6.03 (1H, s, 3-H), 5.91 (1H, s. OH), 1.67 (9H, s, *t*-Bu-H), 1.42 (9H, s, *t*-Bu-H). ^13^C{1 H} NMR (150 MHz, CDCl_3_): 166.3 (1), 161.9 (1), 152.9 (1), 146.5 (1), 138.1 (1), 136.6 (1), 125.8 (1), 80.8 (1), 56.1 (1), 51.9 (1), 28.5 (3), 28.1 (3). Anal. Calcd. for C_16_H_23_N_3_O_3_: C, 62.93; H, 7.59; N, 13.76. Found C, 62.96: H, 7.56; N, 13.78. HRMS (+ESI): *m*/*z* calcd for C_16_H_23_N_3_O_3_ [M + H]^+^ 328.1637; found 328.1640.

2-(*tert*-butyl)-7-(*tert*-butylcarbamoyl)-1-oxo-2,3-dihydro-1*H*-pyrrolo[3,4-*c*]pyridin-3-yl *tert*-butylcarbamate (**10**) White powder (0.092 g, 25% yield). R*_f_* = 0.53 (CH_2_Cl_2_: ethyl acetate 7:3) Mp: 188–190 °C. IR (KBr, cm^−1^): 1689, 1676, 1652 (C=O). ^1^H NMR (600 MHz, CDCl_3_): *δ* 10.52 (1H, s, N-H), 9.63 (1H, s, 6-H), 8.97 (1H, s, 4-H), 7.10 (1H, s, 3-H), 4.75 (1H, s. OH), 1.57 (9H, s, *t*-Bu-H), 1.50 (9H, s, *t*-Bu-H), 1.36 (9H, s, *t*-Bu-H). ^13^C{1 H} NMR (150 MHz, CDCl_3_): 168.3 (1), 161.4 (1), 154.6 (1), 152.8 (1), 147.2 (1), 136.4 (1), 135.8 (1), 126.2 (1), 80.5 (1), 56.3 (1), 51.7 (1), 51.2 (1), 28.7 (3), 28.5 (3), 27.8 (3). Anal. Calcd. for C_21_H_32_N_4_O_4_: C, 62.35; H, 7.97; N, 13.85. Found C, 62.38; H, 7.94; N, 13.88. HRMS (+ESI): *m*/*z* calcd for C_21_H_32_N_4_O_4_ [M + H]^+^ 405.2502; found 405.2508.

*N*,2-di-*tert*-butyl-3-hydroxy-1-oxo-2,3-dihydro-1*H*-pyrrolo[3,4-*c*]pyridine-7-carbothioamide (**9e**). Yellow powder (0.067 g, 21% yield). R*_f_* = 0.27 (CH_2_Cl_2_: ethyl acetate 7:3). Mp: 182–184 °C. IR (KBr, cm^−1^): 1667 (C=O). ^1^H NMR (600 MHz, CDCl_3_): 10.76 (1H, s, N-H), 9.21 (1H, s, 6-H), 8.26 (1H, s, 4-H), 5.98 (1H, s, 3-H), 1.65 (9H, s, *t*-Bu-H), 1.62 (9H, s, *t*-Bu-H). ^13^C{1 H} NMR (150 MHz, CDCl_3_): 188.1 (1), 165.8 (1), 153.7 (1), 144.0 (1), 137.7 (1), 134.0 (1), 132.0 (1), 80.7 (1), 56.8 (1), 56.0 (1), 28.1 (3), 27.2 (3). Anal. Calcd for C_16_H_23_N_3_O_2_S: C, 59.79; H, 7.21; N, 13.07; S, 9.97. Found C, 59.86: H, 7.16; N, 13.04; S, 9.94. HRMS (+ESI): *m*/*z* calcd for C_16_H_23_N_3_O_2_S [M + H]^+^ 322.1589; found 322.1595.

2-(*tert*-butyl)-3-hydroxy-7-(hydroxy(4-methoxyphenyl)methyl)-2,3-dihydro-1*H*-pyrrolo[3,4-*c*]pyridin-1-one (**9f**), was obtained as a mixture of diastereoisomers with a ratio of 1 to 1.4. White powder (0.143 g, 41% yield). R*_f_* = 0.14 (hexane: ethyl acetate 3:7). Mp: 75–80 °C. IR (KBr, cm^−1^): 1696, 1667 (C=O). ^1^H NMR (600 MHz, CDCl_3,_ {d—dominant diastereoisomer, m—minor diastereoisomer}): 8.67 (1Hd, s, 6-H), 8.55 (1Hm, s, 6-H), 8.23 (1Hm, s, 4-H), 8.20 (1Hd, s, 4-H), 7.16 (2Hm, pd, *J* = 8.4 Hz, 2,6-Ar-H), 7.11 (2Hd, pt, *J* = 8.4 Hz, 2,6-Ar-H), 6.82 (2Hm, pd, *J* = 8.4 Hz, 3,5-Ar-H), 6.78 (2Hd, pt, *J* = 8.4 Hz, 3,5-Ar-H), 6.11 (1Hd, s, 3-H), 6.07 (1Hm, s, 3-H), 5.76–5.00 (br. s, overlapping with singlet 5.40, OH), 3.77 (3Hm, s, OMe-H), 3.73 (3Hd, s, OMe-H),1.62 and 1.61 two overlapping singlet of m and d (9H, s, *t*-Bu-H). ^13^C{1 H} NMR (150 MHz, CDCl_3_):167.3 (1d), 167.1 (1m), 159.2 (1m), 159.0 (1d), 148.9 (1d), 148.7 (1m), 143.4 (1d), 142.9 (1m), 139.7 (1d), 139.1 (1m), 137.6 (1d and 1m), 137.1 (1d and 1m), 136.4 (1d and 1m), 134.5 (1d and 1m), 133.3 (1d and 1m), 127.9 (2m), 127.3 (2d), 114.0 (2m), 113.9 (2d), 82.5 (1d), 81.8 (1m), 73.1 (1d), 71.3 (1m), 56.0 (1d), 55.9 (1m), 55.3 (1m), 55.2 (1d), 28.3 (1m), 28.2 (1d). Anal. Calcd for C_19_H_22_N_2_O_4_: C, 66.65; H, 6.48; N, 8.18. Found C, 66.79: H, 6.29; N, 8.24; S, 9.94. HRMS (+ESI): *m*/*z* calcd for C_19_H_22_N_2_O_4_ [M + H]^+^ 343.1658; found 343.1664.

#### 3.2.3. Transformation of Hydroxylactam **9c** and **9d** into Imides **11** and **12**

A solution of Jones reagent (0.39 mmol of chromium trioxide dissolved in 3 mL of sulfuric acid end water 5:2 *v*/*v*) was added slowly to a solution of hydroxylactam **9c** or **9d** (0.3 mmol, 98 mg, 92 mg, respectively) in acetone (10 mL). The reaction mixture was stirred at room temperature for 4 h, the solid was filtered off and the filtrate was stirred with excess solid sodium bicarbonate. The solid was filtered off and washed with acetone (5 mL). Evaporation of the solvent and column chromatography (silica gel, CH_2_Cl_2_:ethyl acetate 7:3) were afforded imide **11** as a yellow powder (93 mg, 96%) or **12** as a white powder (90 mg, 99%).

2-(*tert*-butyl)-1,3-dioxo-*N*-phenyl-2,3-dihydro-1*H*-pyrrolo[3,4-*c*]pyridine-7-carboxamide (**11**). Yellow powder (0.302 g, 93% yield). R*_f_* = 0.32 (CH_2_Cl_2_: ethyl acetate 7:3). Mp: 220–221 °C. IR (KBr, cm^−1^): 1699, 1662, 1629 (C=O). ^1^H NMR (600 MHz, CDCl_3_): 11.81 (1H, s, N-H), 9.92 (1H, s, 4-H), 9.20 (1H, s, 6-H), 7.81 (2H, pd, *J* = 7.8 Hz, 2,6-Ph-H), 7.39 (2H, pt, *J* = 7.8 Hz, 3,5-Ph-H), 7.17 (1H, pt, *J* = 7.8 Hz. 4-PhH), 1.77 (9H, s, *t*-Bu-H). ^13^C{1 H} NMR (150 MHz, CDCl_3_): 171.0 (1), 167.1 (1), 159.2 (1), 159.0 (1), 146.9 (1), 138.0 (1), 135.1 (1), 129.1 (2), 124.9 (1), 120.4 (2), 60.1 (1), 28.9 (3). Anal. Calcd for C_18_H_17_N_3_O_3_: C, 66.86; H, 5.30; N, 13.00. Found 66.90: H, 5.25; N, 14.89. HRMS (+ESI): *m*/*z* calcd for C_18_H_17_N_3_O_3_ [M + H]^+^ 324.1348; found 324.1357.

*N*,2-di-*tert*-butyl-1,3-dioxo-2,3-dihydro-1*H*-pyrrolo[3,4-*c*]pyridine-7-carboxamide (**12**) White solid (0.250 g, 82% yield). R*_f_* = 0.79 (CH_2_Cl_2_: ethyl acetate7:3). Mp: 104–105 °C. IR (KBr, cm^−1^): 1775, 1695, 1656 (C=O). ^1^H NMR (600 MHz, CDCl_3_): 9.80 (1H, s, 4-H), 9.59 (1H, s, N-H), 9.15 (1H, s 6-H), 1.71 (9H, s, *t*-Bu-H), 1.52 (9H, s, *t*-Bu-H). ^13^C{1 H} NMR (150 MHz, CDCl_3_):170.5 (1), 167.3 (1), 160.2 (1), 158.7 (1), 146.2 (1), 135.0 (1), 125.9 (1), 124.9 (1), 59.7 (1), 52.1 (1), 28.9 (3), 28.5 (3). Anal. Calcd for C_16_H_21_N_3_O_3_: C, 63.35; H, 6.98; N, 13.85. Found 63.39: H, 6.93; N, 13.89. HRMS (+ESI): *m*/*z* calcd for C_16_H_21_N_3_O_3_ [M + H]^+^ 304.1661; found 304.1666.

## 4. Quantum Chemical Calculations

The DFT computations were performed using the ωB97xD/6-311+G(d,p) level of theory implemented in the Gaussian package [69,70]. The computational PlGrid infrastructure at the polish “Cyfronet” center was utilized. For the calculations, we used the 6-311+G(d,p) basis set according to suggestions published earlier regarding the substitution reaction with the participation of lithium-organic segments [43]. All localized stationary points were verified on the basis of the full vibrational analysis. We found that starting molecules, pre-reaction complexes and products had positive Hessian matrices. On the other hand, the optimized transition state (**TS**) exhibits one negative eigenvalue in the Hessian matrices. Next, the IRC trajectories confirmed, without doubt, its postulated nature and the role within the energy profile. The presence of solvents (THF) in the reaction environment was included using the IEFPCM (Integral Equation Formalism Polarizable Continuum Model) algorithm [71]. Calculations of all critical structures were performed at a temperature T = 298 K and pressure *p* = 1 atm. The same methodology has already been successfully and very recently applied to explore the mechanistic aspects of various substitution processes.

For optimized stationary structures of reagents, the reactivity descriptors were estimated used universal equations recommended in the literature. In particular, global nucleophilicity (N) [72] was expressed using the equation:

N = E_HOMO_ − E_HOMO(tetracyanoethene)_

The local nucleophilicities (N_k_) condensed to atom *k* was calculated using global nucleophilicity (N) and *Parr* functions P^−^_k_ [73] according to the following formula:

N_k_ = P^−^_k_·N

## 5. Conclusions

The present method is quite useful for the functionalization of the seventh-position of 2-(*tert*-butyl)-3-hydroxy-2,3-dihydro-1*H*-pyrrolo[3,4-*c*]pyridin-1-one (**7**). The reaction protocol is simple and occurs under mild conditions. All obtained products are stable, pure, crystalline and can be easily identified based on spectral data and X-ray analysis results. The observed regioselectivity of the lithiation process can be explained by both the distribution of local reactivities and thermodynamic factors. Both factors operate concurrently. It should be emphasized that the process of the replacement of a lithium atom at the heterocyclic carbon atom for an electrophile segment does not proceed in a manner analogous to standard substitution processes. DFT calculation data shed light on the nature of the transition state. The described methodology and reaction mechanism can be applied to a larger group of reagents differing in the structure of the introduced functional group.

## Figures and Tables

**Table 1:** Lithiation of the hydroxylactam **7** and the reaction of generated lithiated reactive intermediated with electrophiles ^(a),(b),(c)^.

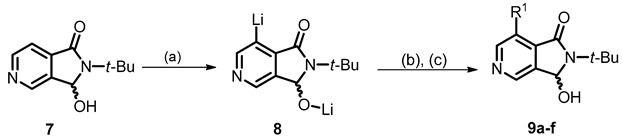				
Entry	Source of the Electrophile	R^1^	Yield 9 ^(d)^ [%]	Yield [%] Recovered 7
entry 1	MeOD	D	**9a**, ~100 ^(e)^	0
entry 2	SiMe_3_Cl	SiMe_3_	**9b**, 70	15
entry 3	PhNCO	CONHPh	**9c**, 57	35
entry 4	*t*-BuNCO	CONH(*t*-Bu)	**9d**, 73	0
entry 5	*t*-BuNCS	CSNH(*t*-Bu)	**9e**, 21	40 ^(f)^
entry 6	4-methoxybenzaldehyde	CH(OH)C_6_H_4_-4-OMe	**9f**, 41	31 ^(c)^

^(a)^ *n*-BuLi (2 mmol), TMEDA (2 mmol), THF, t = 1 h, −78 °C, argon atmosphere. ^(b)^ Electrophile (2 mmol), t = 1.5 h, −78 °C: MeOD, SiMe_3_Cl, PhNCO. *t*-BuNCO, *t*-BuNCS, 4-methoxybenzaldehyde, ^(c)^ sat. aq. NH_4_Cl. ^(d)^ All yields represented pure materials. ^(e)^ Data represented yield identified by ^1^H NMR. ^(f)^ In the case of entries 5 and 6, an additional unidentified mixture was isolated.

**Table 2:** Thermodynamic function of the formation of isomeric lactams **8″** and **8″′**, calculated in relation to the analogous function for **8′** according to the ωB97xD/6-311+G(d,p) (PCM) calculations.

Compounds	∆H [kcal/mol]	∆S [cal/molK]	∆G [kcal/mol]
**8′**	0.00	0.00	0.00
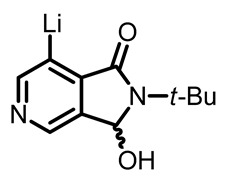			
**8″**	8.32	2.7	7.51
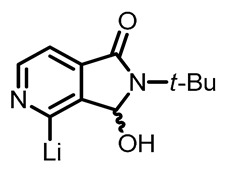			
**8″′**	10.71	3.7	9.62
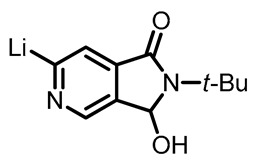			

## Data Availability

Data are contained within the article.

## Supplementary Materials

The following supporting information can be downloaded at https://www.mdpi.com/article/10.3390/molecules31111973/s1, Figures S1–S25, description of Rtg analyses and DFT computational data. Key spectral characteristics of products (^1^H NMR and ^13^ CNMR spectra of **7**, **9a**–**f**, **10**–**12**), key geometries of optimized structures, X-ray Diffraction Analysis. Table S1. Crystal structure, data collection and refinement parameters.
