# Supplementary material for: On the Question of the Full Selective Synthesis of Potentially Bioactive of 2-(tert-Butyl)-3-hydroxy-7-2,3-dihydro-1H-pyrrolo[3,4-c]pyridin-1-ones and Their Derivatives: Experimental and DFT Computational Study
Source: Molecules. 2026 Jun 5;31(11):1973. doi: 10.3390/molecules31111973 (PMC13258793; doi:10.3390/molecules31111973)
Supplement: Supplementary file 1 [file molecules-31-01973-s001.zip › molecules-4333188-supplementary.pdf]

SUPPLEMENTARY MATERIAL

On the question of the full selective synthesis of potentially bioactive of 2-(*tert*-butyl)-3-hydroxy-7-2,3-dihydro-1*H*-pyrrolo[3,4-c]pyridin-1-ones and their derivatives: experimental and DFT computational study

Magdalena Ciechańska[1]*, Ewelina Wielgus[2], Rafał Dolot[2],
Andrzej Jóźwiak[1], Radomir Jasiński[3]*

[1] Key spectral characteristics of products

[1a] ^{1}HNMR and ^{13}CNMR spectra of **7**, **9a-f**, **10 -12**……………………………………...1

[2] Key geometries of optimized structures…………………………………………………12

[3] X-ray Diffraction Analysis……………………………………………………………18

[1] <u>Key spectral characteristics of products</u>

[1a] ^{1}HNMR and ^{13}CNMR spectra of **7**, **9a-f**, **10 -12.**

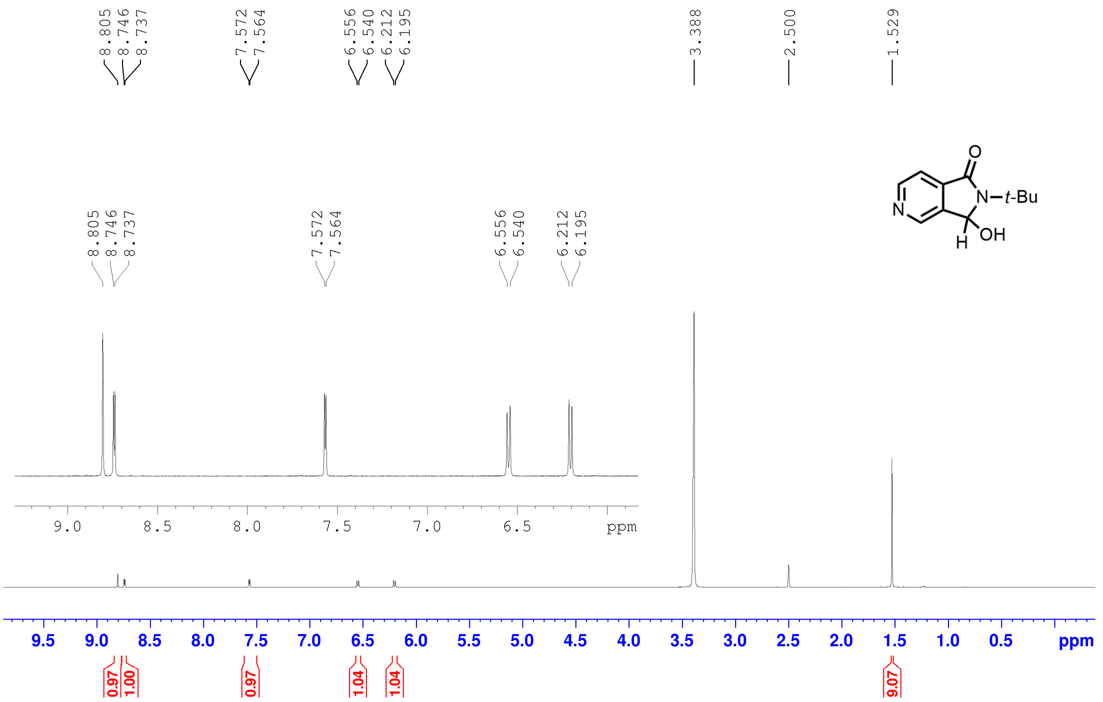

Figure S1. ^{1}H NMR spectrum of compound **7** (600 MHz, DMSO-d_6, room temperature).

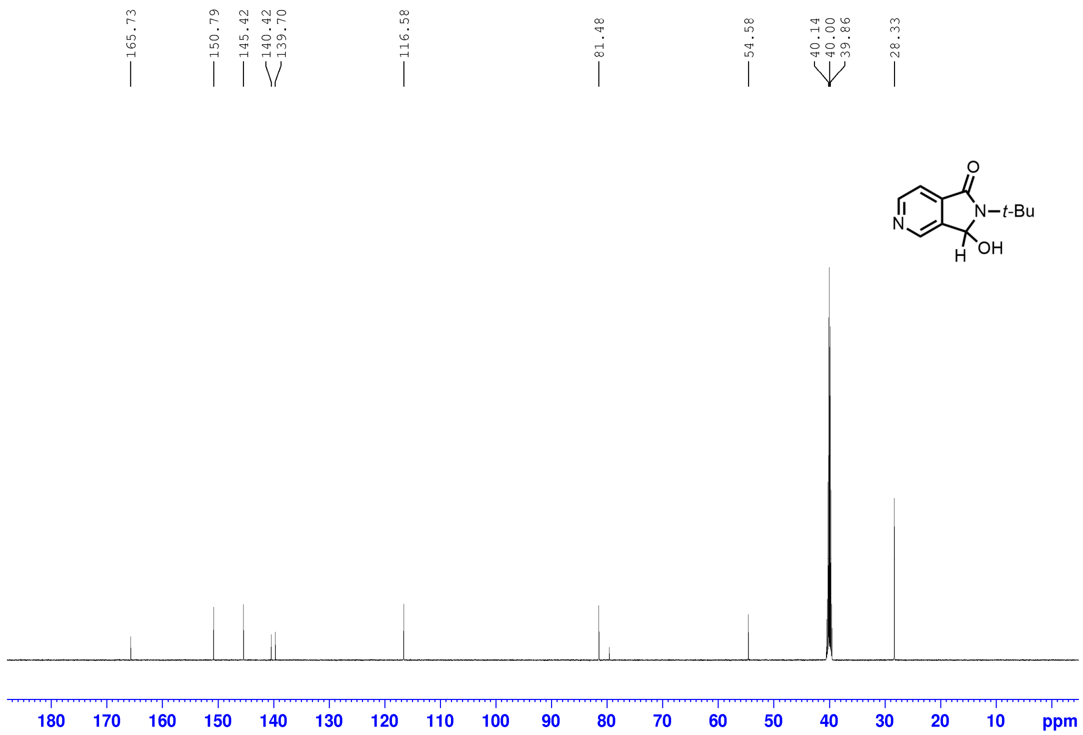

Figure S2. ^{13}C NMR spectrum of compound **7** (150 MHz, DMSO-d_6, room temperature).

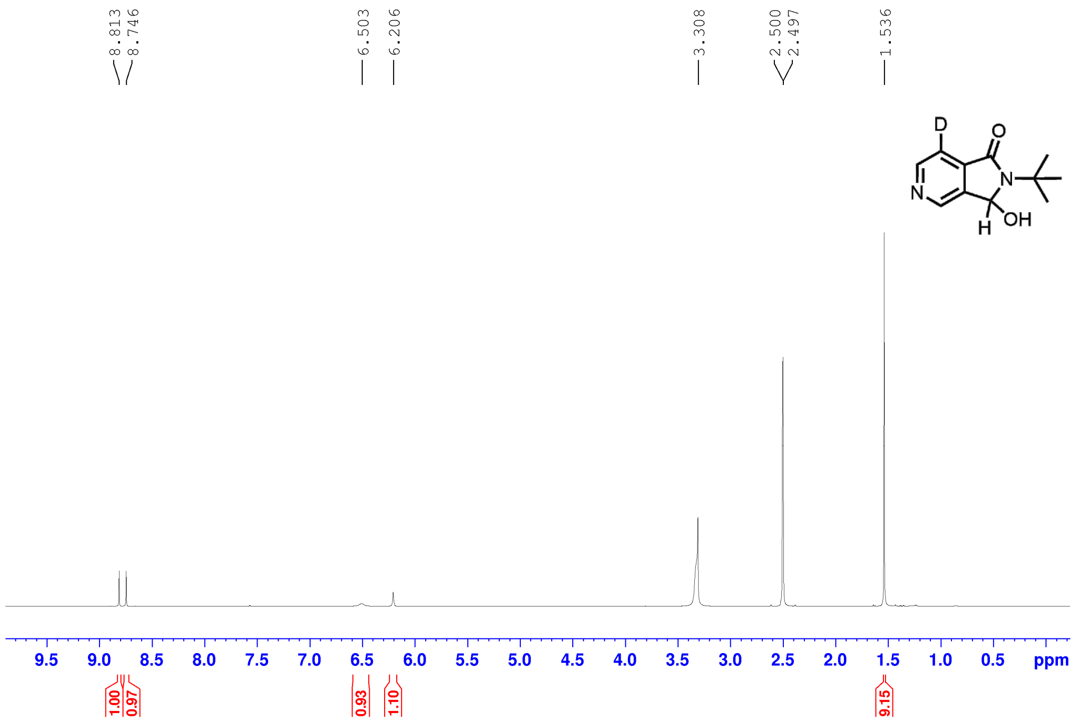

Figure S3. ^{1}H NMR spectrum of compound **9a** (600 MHz, DMSO-d_6, room temperature).

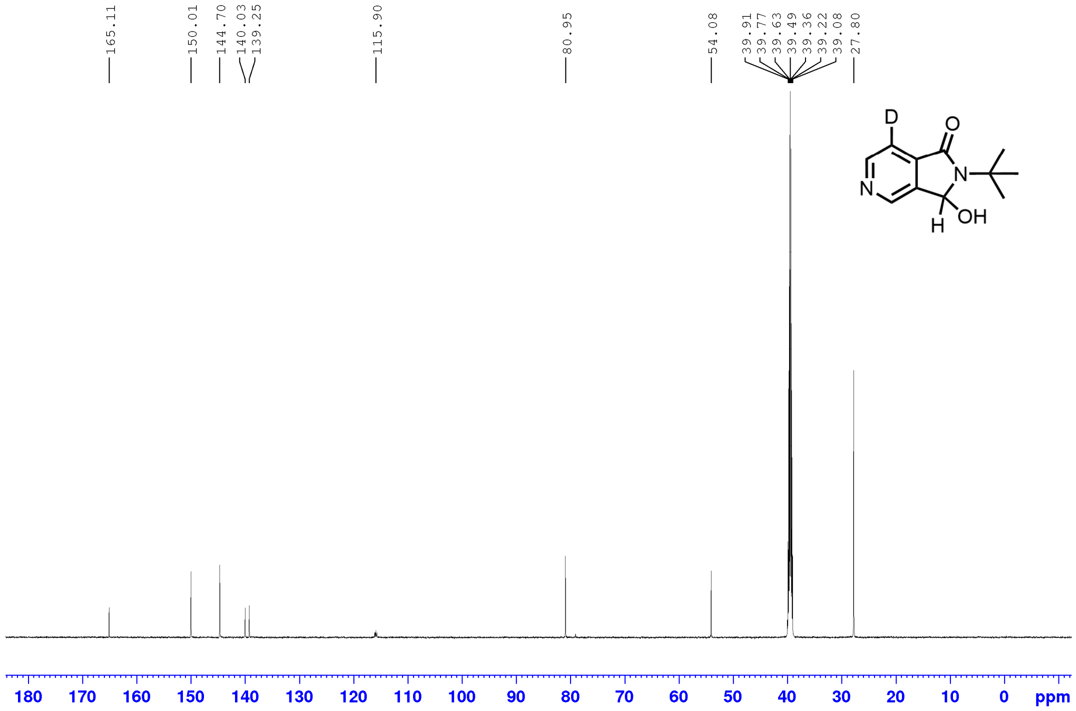

Figure S4. ^{13}C NMR spectrum of compound **9a** (150 MHz, DMSO-d_6, room temperature).

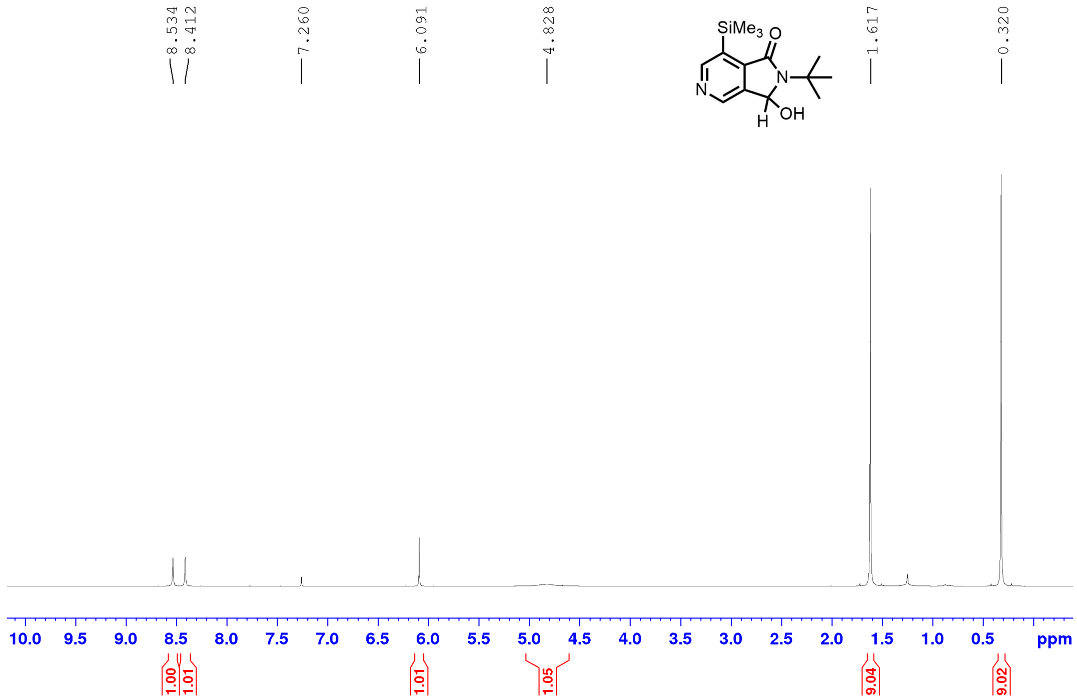

Figure S5. ^{1}H NMR spectrum of compound **9b** (600 MHz, $CDCl_3$, room temperature).

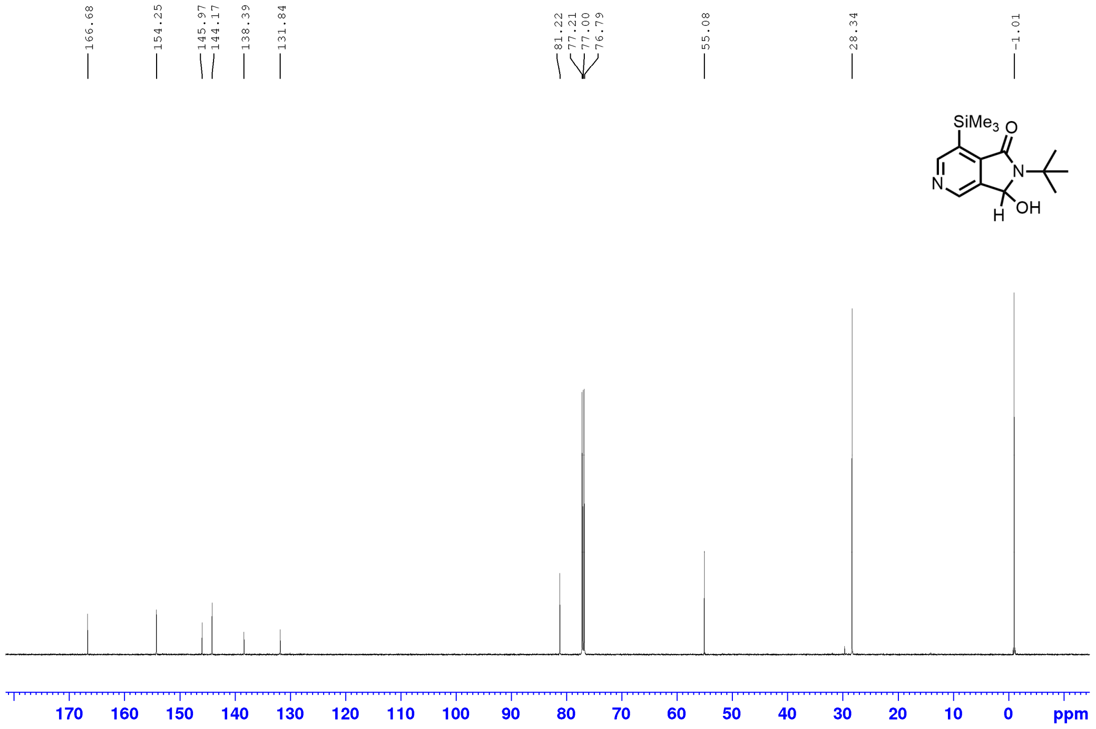

Figure S6. ^{13}C NMR spectrum of compound **9b** (150 MHz, $CDCl_3$, room temperature).

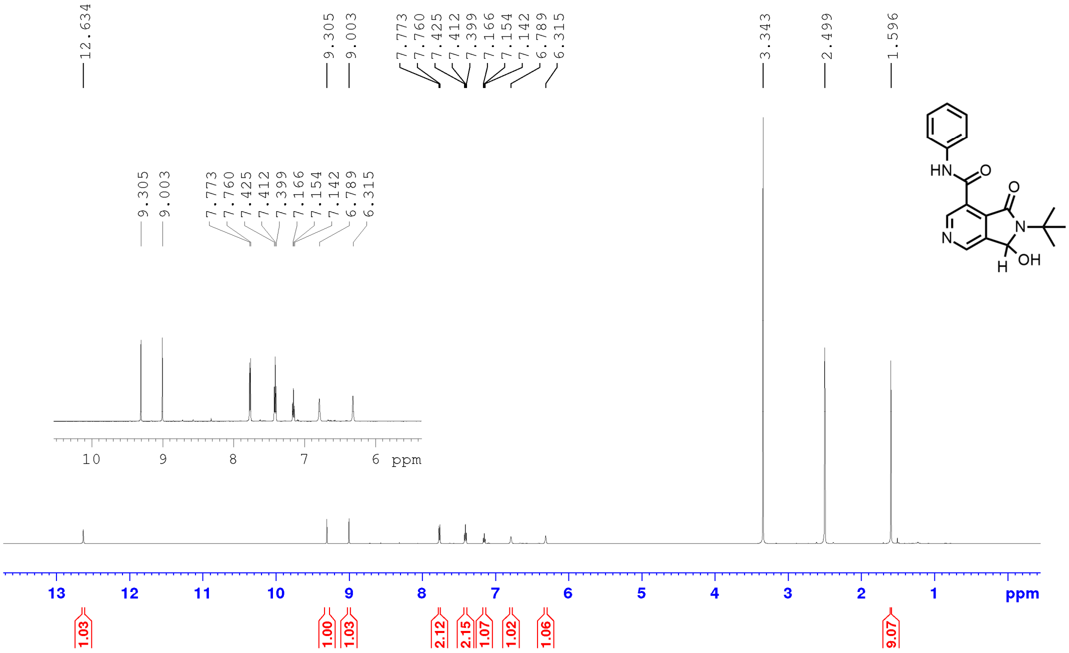

Figure S7. ^{1}H NMR spectrum of compound **9c** (600 MHz, DMSO-d_6, room temperature).

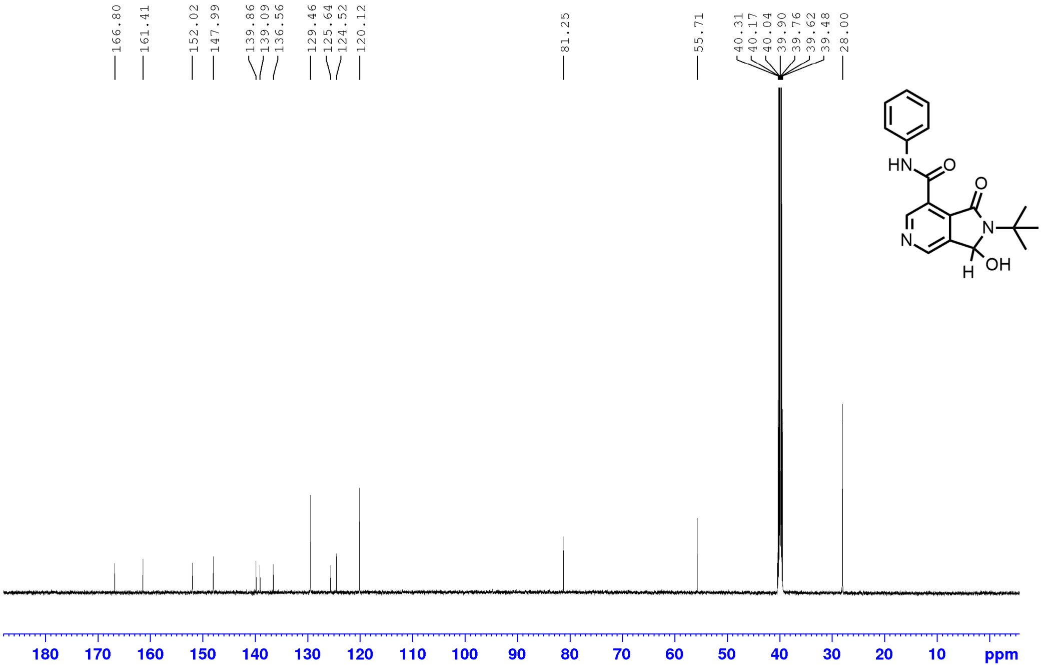

Figure S8. ^{13}C NMR spectrum of compound **9c** (150 MHz, DMSO-d_6, room temperature).

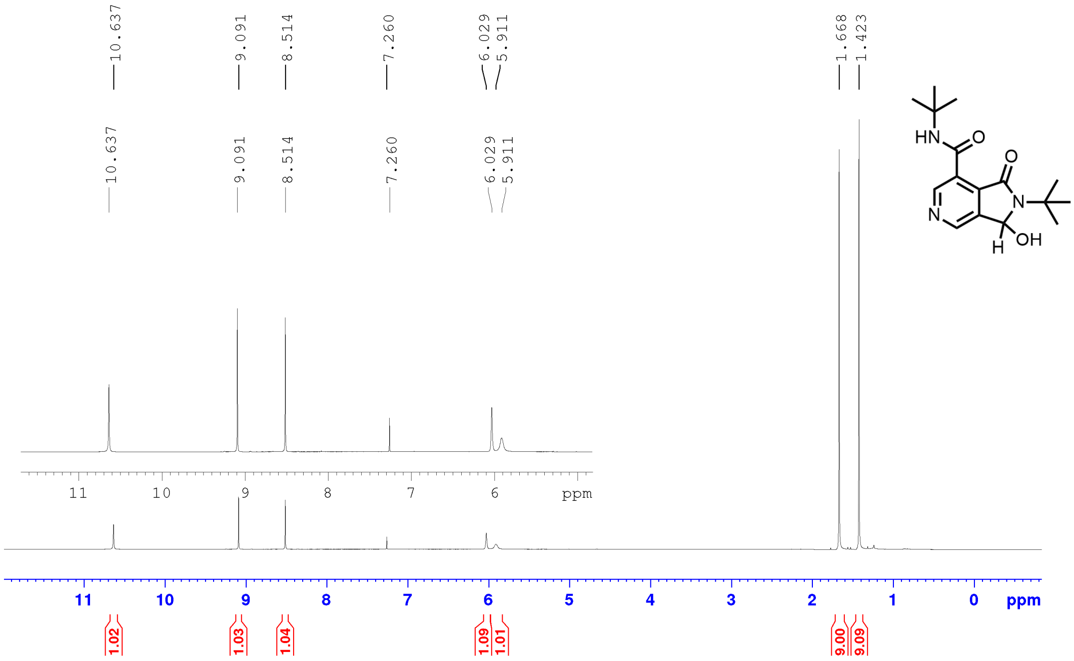

Figure S9. ^{1}H NMR spectrum of compound **9d** (600 MHz, $CDCl_3$, room temperature).

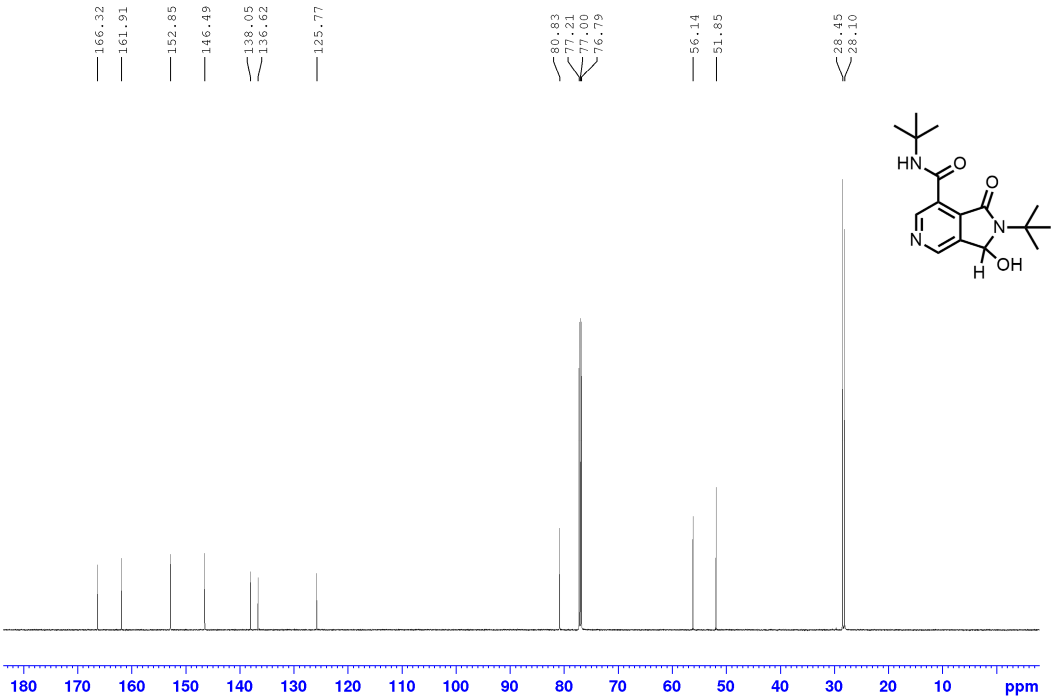

Figure S10. ^{13}C NMR spectrum of compound **9d** (150 MHz, $CDCl_3$, room temperature).

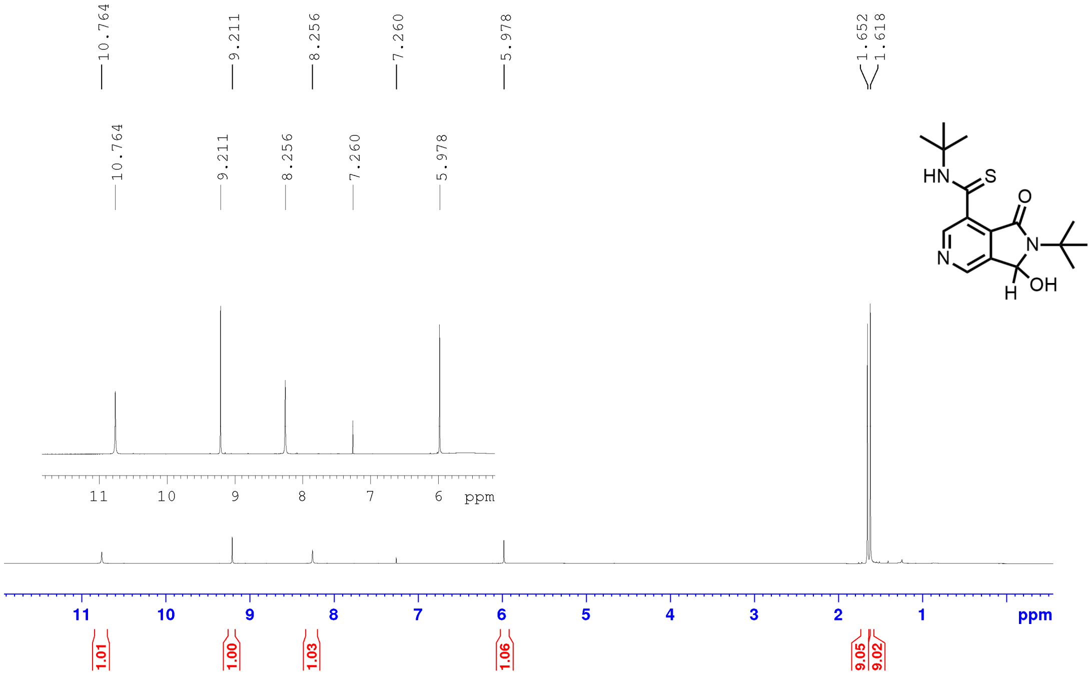

Figure S11. ^{1}H NMR spectrum of compound **9e** (600 MHz, $CDCl_3$, room temperature).

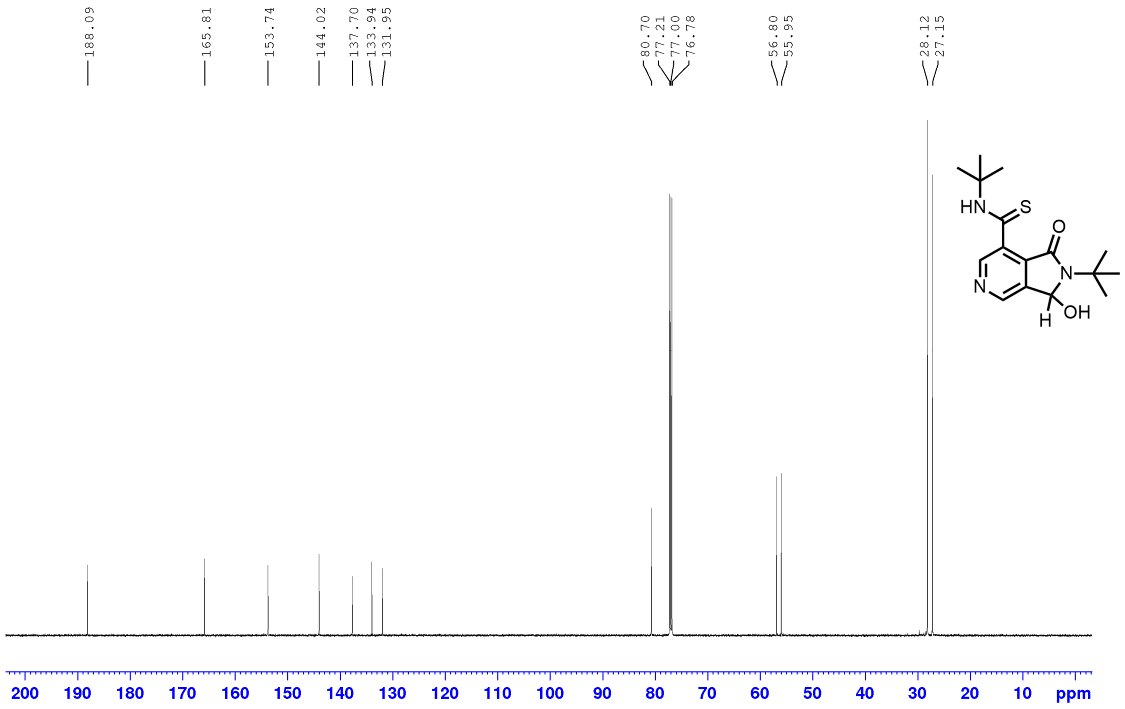

Figure S12. ^{13}C NMR spectrum of compound **9e** (150 MHz, $CDCl_3$, room temperature).

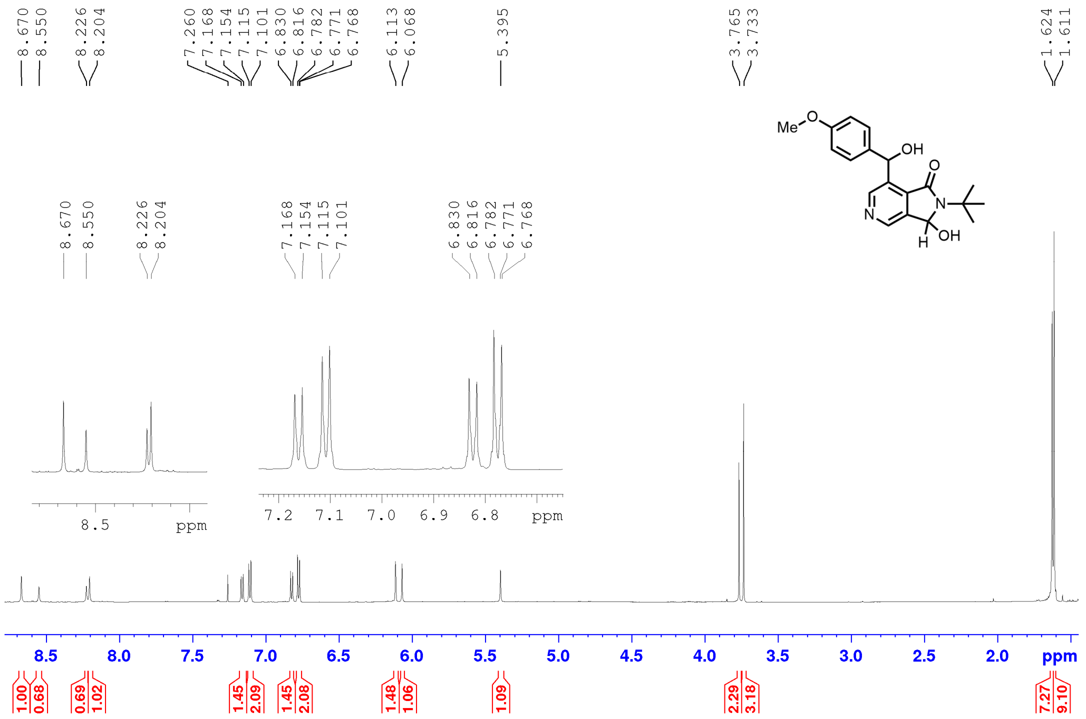

Figure S13. ^{1}H NMR spectrum of compound **9f** (600 MHz, $CDCl_3$, room temperature).

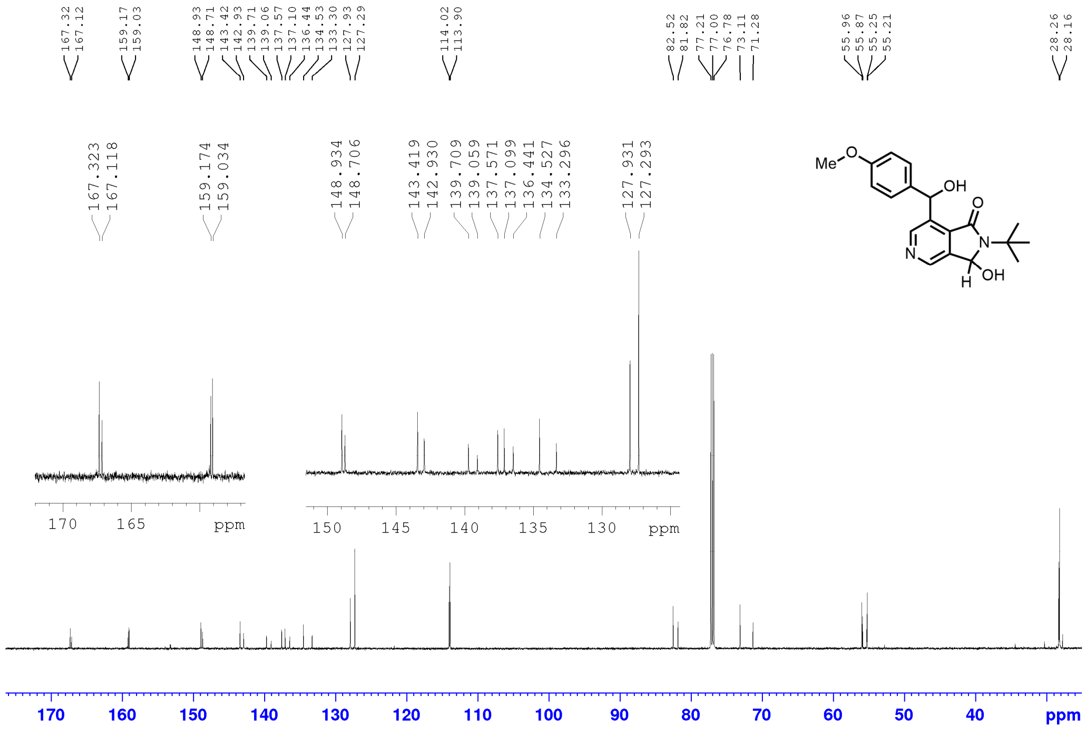

Figure S14. ^{13}C NMR spectrum of compound **9f** (150 MHz, $CDCl_3$, room temperature).

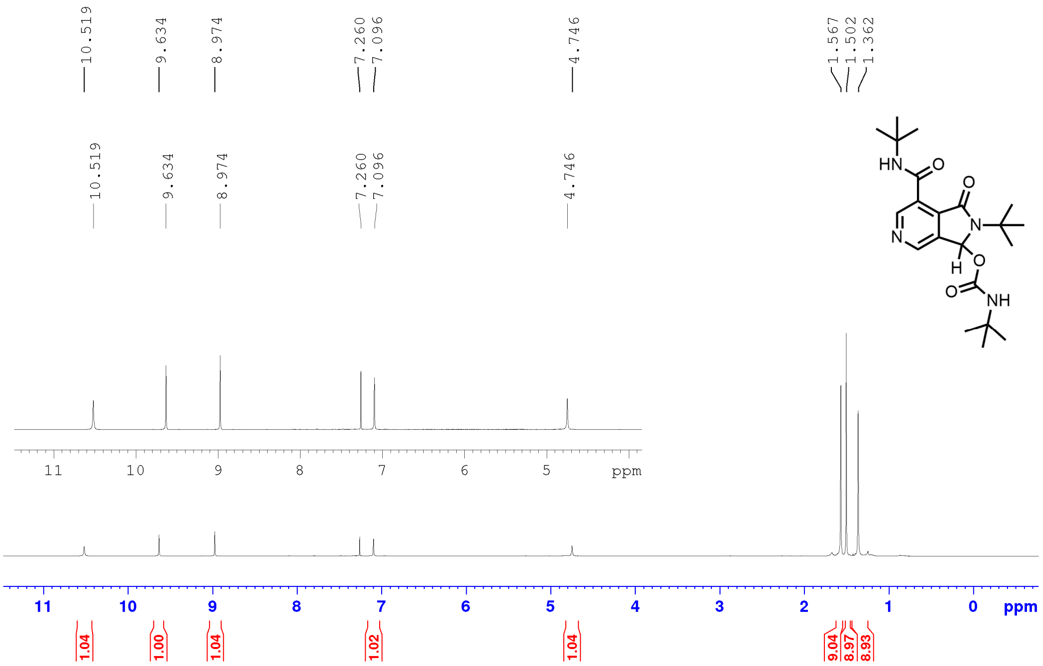

Figure S15. ^{1}H NMR spectrum of compound **10** (600 MHz, $CDCl_3$, room temperature).

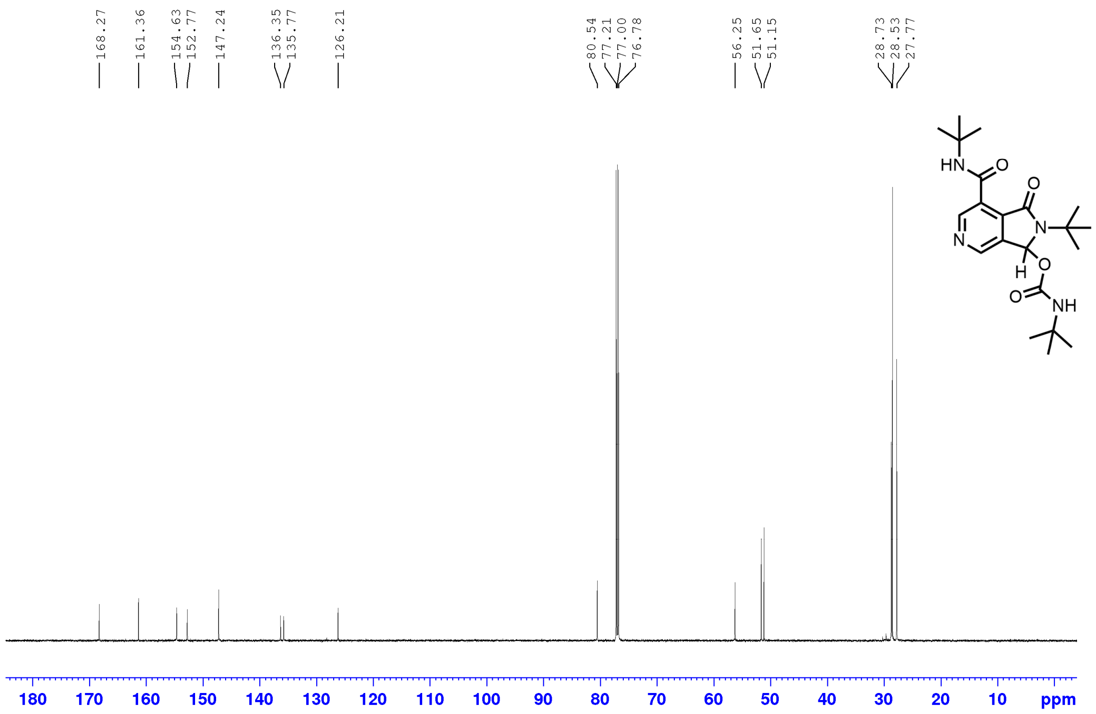

Figure S16. ^{13}C NMR spectrum of compound **10** (150 MHz, $CDCl_3$, room temperature).

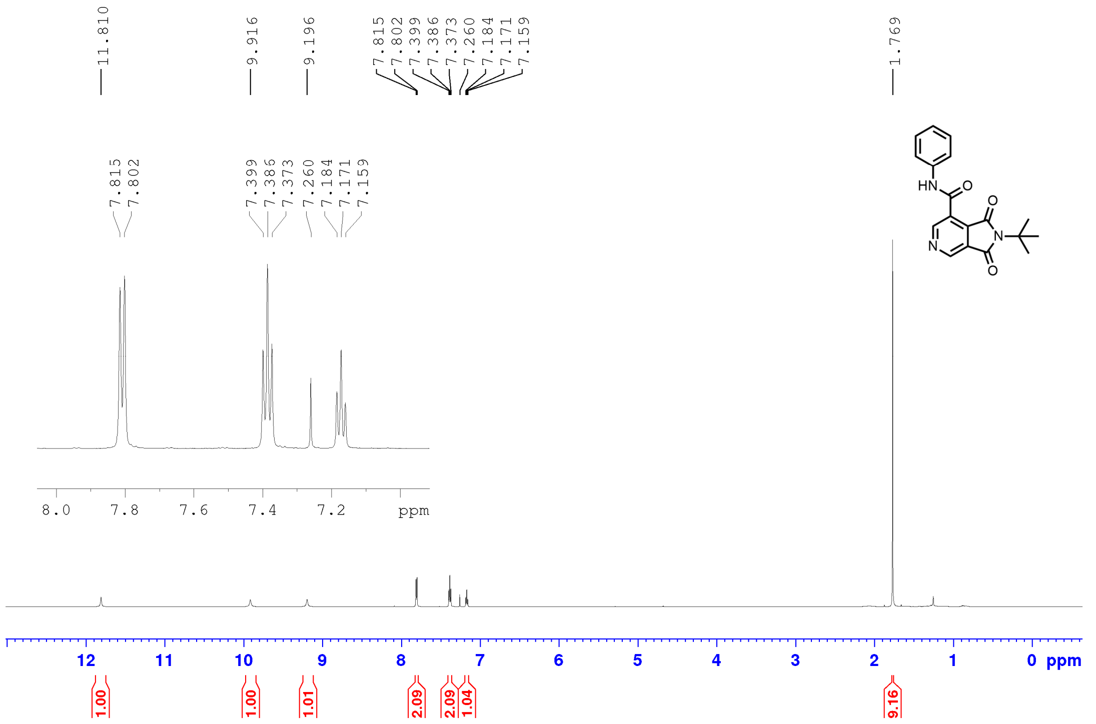

Figure S17. ^{1}H NMR spectrum of compound **11** (600 MHz, $CDCl_3$, room temperature).

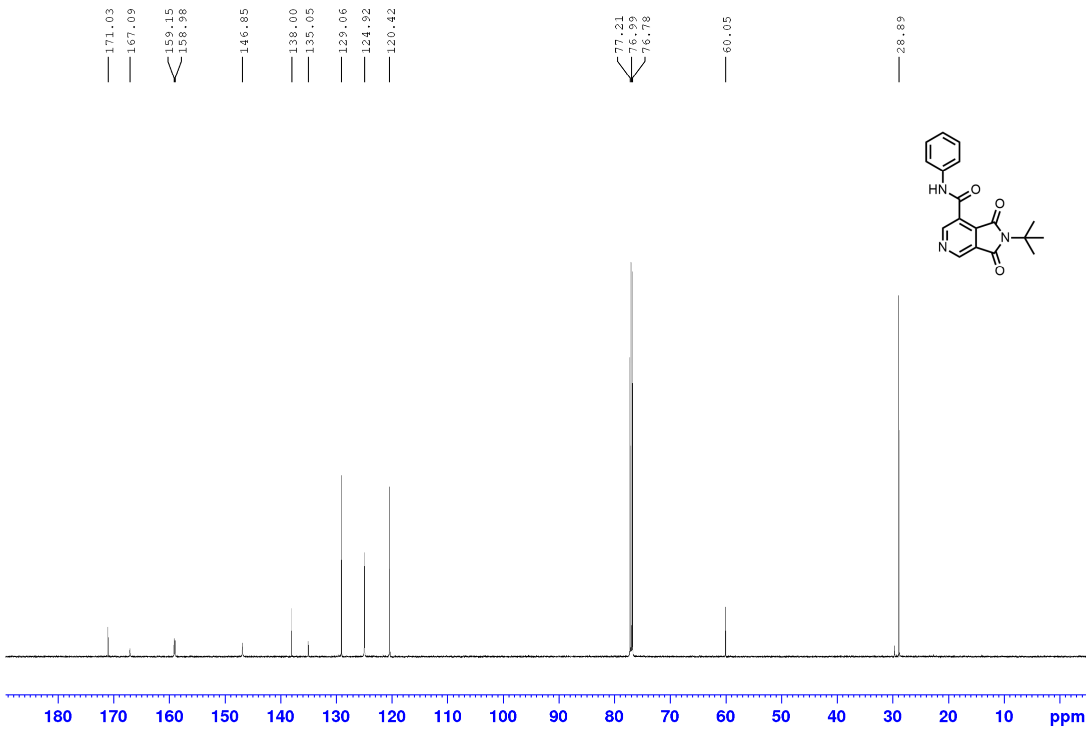

Figure S18. ^{13}C NMR spectrum of compound **11** (150 MHz, $CDCl_3$, room temperature).

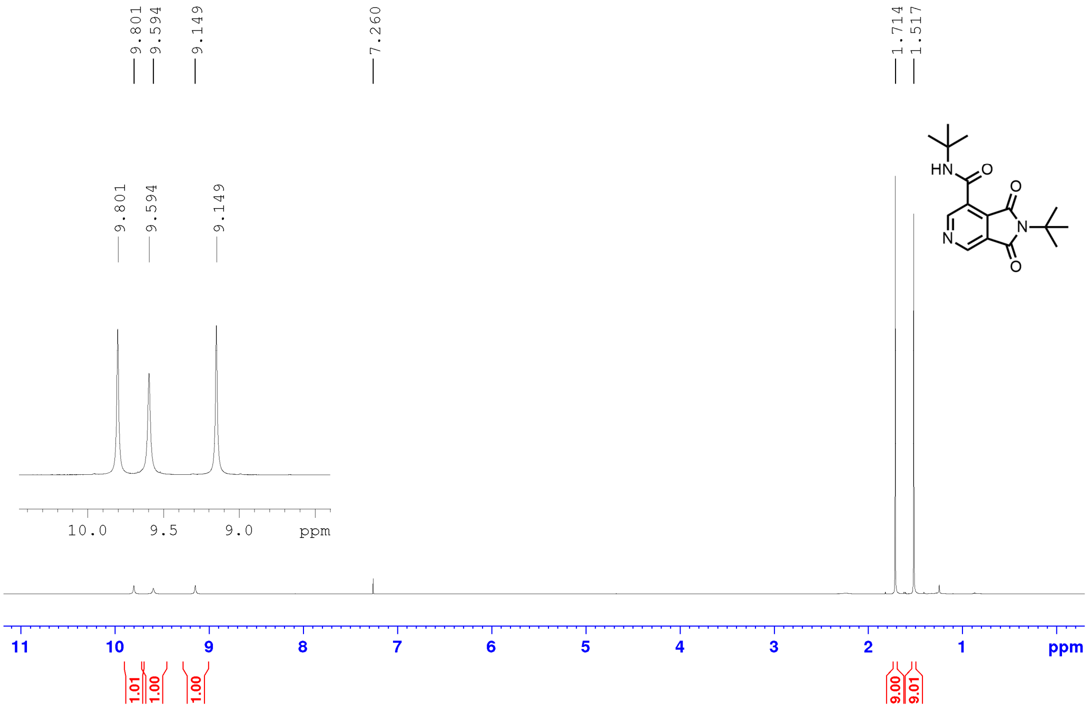

Figure S19. ^{1}H NMR spectrum of compound **12** (600 MHz, $CDCl_3$, room temperature).

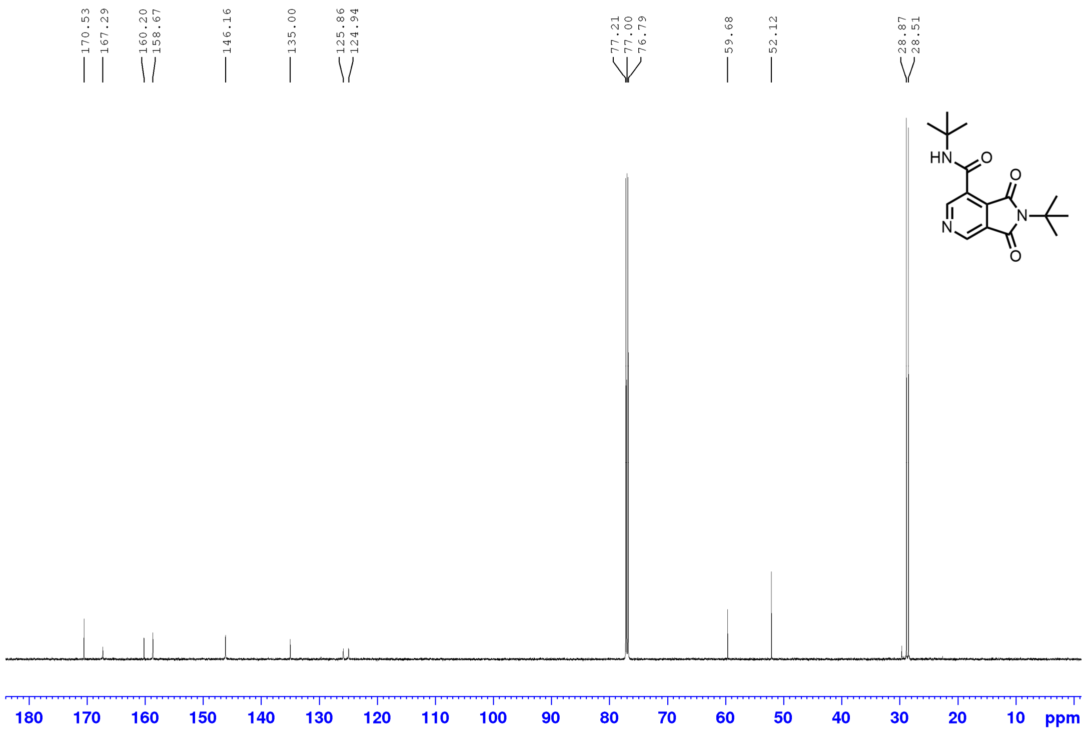

Figure S20. ^{13}C NMR spectrum of compound **12** (150 MHz, $CDCl_3$, room temperature).

[2] Key geometries of optimized structures

7

Center Number	Atomic Number	Atomic Type	Coordinates (Angstroms) X	Y	Z
1	6	0	-1.335154	-0.702445	-0.064079
2	6	0	-1.365895	0.656407	0.153199
3	6	0	-2.585057	1.298180	0.280088
4	6	0	-3.685937	-0.702877	0.014396
5	6	0	0.089540	-1.134282	-0.165492
6	1	0	-2.654176	2.370036	0.436599
7	1	0	-4.645145	-1.208963	-0.029552
8	7	0	-3.732973	0.619181	0.214883
9	8	0	0.480298	-2.281308	-0.288387
10	6	0	0.034939	1.200810	0.174810
11	6	0	2.327772	-0.043324	0.118154
12	6	0	2.959357	-0.743661	-1.091331
13	1	0	4.047737	-0.722996	-0.991640
14	1	0	2.635105	-1.780417	-1.163271
15	1	0	2.687286	-0.223148	-2.013121
16	6	0	2.652071	-0.787972	1.420137
17	1	0	3.734953	-0.835574	1.559915
18	1	0	2.218221	-0.265645	2.277640
19	1	0	2.263259	-1.806368	1.393916
20	6	0	2.904193	1.374421	0.201834
21	1	0	3.973689	1.293827	0.404491
22	1	0	2.796756	1.912221	-0.742928
23	1	0	2.470157	1.959789	1.016960
24	7	0	0.848921	0.000735	-0.054524
25	8	0	0.154275	2.177476	-0.827718
26	1	0	0.894318	2.753037	-0.625610
27	1	0	0.279325	1.628262	1.152733
28	6	0	-2.507966	-1.429202	-0.136417
29	1	0	-2.516892	-2.499150	-0.301602

Pyridine

Center Number	Atomic Number	Atomic Type	Coordinates (Angstroms) X	Y	Z
1	6	0	-1.138059	-0.721902	0.000000
2	6	0	1.141632	-0.716422	-0.000001
3	6	0	1.192305	0.672239	0.000000
4	1	0	-2.053481	-1.306440	-0.000001
5	1	0	2.059220	-1.297478	-0.000000
6	1	0	2.147944	1.182208	0.000001
7	7	0	0.003185	-1.413581	-0.000002
8	6	0	-1.195351	0.667149	0.000001

9	6	0	-0.003317	1.379138	0.000000
10	1	0	-2.153961	1.172068	0.000002
11	1	0	-0.005275	2.463496	0.000002

8’

Center Number	Atomic Number	Atomic Type	Coordinates (Angstroms) X	Y	Z
1	6	0	1.355603	0.571561	-0.042628
2	6	0	1.336808	-0.798021	0.147115
3	6	0	2.545525	-1.453060	0.261152
4	6	0	3.640060	0.573543	0.034893
5	6	0	-0.050097	1.027358	-0.138907
6	1	0	2.619190	-2.528243	0.398129
7	1	0	4.628633	1.033621	0.010112
8	7	0	3.691609	-0.760930	0.204543
9	8	0	-0.389898	2.210653	-0.249937
10	6	0	-0.081591	-1.295767	0.165763
11	6	0	-2.337114	0.041314	0.119864
12	6	0	-2.935392	0.783644	-1.081499
13	1	0	-4.023817	0.813215	-0.983297
14	1	0	-2.561796	1.804648	-1.141013
15	1	0	-2.686430	0.261732	-2.009013
16	6	0	-2.635425	0.782517	1.430263
17	1	0	-3.715870	0.870383	1.570267
18	1	0	-2.222338	0.234113	2.281694
19	1	0	-2.206862	1.785203	1.415971
20	6	0	-2.972392	-1.351672	0.184288
21	1	0	-4.038386	-1.229784	0.385528
22	1	0	-2.882068	-1.882360	-0.766051
23	1	0	-2.563638	-1.963060	0.993043
24	7	0	-0.862087	-0.058633	-0.049308
25	8	0	-0.257093	-2.256797	-0.844863
26	1	0	-1.003377	-2.817345	-0.625315
27	1	0	-0.347849	-1.719826	1.139600
28	6	0	2.489124	1.381290	-0.106733
29	3	0	1.414411	3.289183	-0.279777

8’’

Center Number	Atomic Number	Atomic Type	Coordinates (Angstroms) X	Y	Z
1	6	0	-1.226970	-0.860272	-0.077703
2	6	0	-1.367181	0.495610	0.117372
3	6	0	-2.605314	1.146196	0.257446
4	6	0	0.217964	-1.198902	-0.166560

5	7	0	-3.695319	0.310824	0.199195
6	8	0	0.695294	-2.320262	-0.255712
7	6	0	0.003132	1.124219	0.133055
8	6	0	2.376491	0.037181	0.127422
9	6	0	3.086425	-0.670134	-1.034440
10	1	0	4.168571	-0.579450	-0.906276
11	1	0	2.824385	-1.725818	-1.074319
12	1	0	2.811135	-0.202737	-1.983601
13	6	0	2.712881	-0.632556	1.467318
14	1	0	3.792540	-0.608691	1.637361
15	1	0	2.224059	-0.104202	2.291162
16	1	0	2.385551	-1.672834	1.473634
17	6	0	2.871207	1.488137	0.163995
18	1	0	3.938285	1.478437	0.394648
19	1	0	2.758815	1.977668	-0.806725
20	1	0	2.383410	2.082554	0.941251
21	7	0	0.905590	-0.014159	-0.083174
22	8	0	0.078909	2.106262	-0.878153
23	1	0	0.836543	2.669285	-0.710802
24	1	0	0.220740	1.579921	1.106260
25	6	0	-2.345686	-1.674093	-0.132000
26	1	0	-2.285955	-2.745905	-0.279025
27	3	0	-3.074318	3.205828	0.314843
28	6	0	-3.558896	-1.013047	0.017738
29	1	0	-4.484964	-1.584398	-0.011188

8’’’

Center Number	Atomic Number	Atomic Type	Coordinates (Angstroms) X	Y	Z
1	6	0	-1.245038	-0.631402	-0.071582
2	6	0	-1.218022	0.730109	0.149136
3	6	0	-2.417994	1.399160	0.282092
4	6	0	-3.669509	-0.604556	0.011170
5	6	0	0.160991	-1.112146	-0.176461
6	1	0	-2.451005	2.474930	0.443203
7	7	0	-3.592343	0.755071	0.218783
8	8	0	0.521851	-2.272731	-0.292165
9	6	0	0.200304	1.221367	0.168306
10	6	0	2.440062	-0.109918	0.120311
11	6	0	3.064877	-0.866942	-1.058875
12	1	0	4.152005	-0.882468	-0.942475
13	1	0	2.703057	-1.892110	-1.108740
14	1	0	2.825863	-0.362447	-1.998746
15	6	0	2.714497	-0.832668	1.446476
16	1	0	3.792009	-0.924151	1.606609
17	1	0	2.290099	-0.269509	2.282881
18	1	0	2.280365	-1.833104	1.439911

19	6	0	3.080385	1.282077	0.174633
20	1	0	4.143422	1.159579	0.391012
21	1	0	3.003848	1.799170	-0.784806
22	1	0	2.664330	1.907398	0.969036
23	7	0	0.969798	-0.005336	-0.077559
24	8	0	0.362380	2.207792	-0.825504
25	1	0	1.133338	2.739603	-0.620227
26	1	0	0.473061	1.632924	1.146300
27	3	0	-5.749867	-0.968513	0.001429
28	6	0	-2.447744	-1.307123	-0.139552
29	1	0	-2.453681	-2.380657	-0.309385

MC

Center Number	Atomic Number	Atomic Type	Coordinates (Angstroms) X	Y	Z
1	6	0	0.411684	1.362605	-0.554123
2	6	0	1.252362	1.873798	0.417655
3	6	0	0.989427	3.138094	0.902057
4	6	0	-0.854407	3.264078	-0.490702
5	6	0	0.892798	0.002770	-0.887779
6	1	0	1.608004	3.617805	1.655370
7	1	0	-1.686184	3.905785	-0.782862
8	7	0	-0.065676	3.826073	0.444052
9	8	0	0.311596	-0.768383	-1.660086
10	6	0	2.338084	0.883454	0.731958
11	6	0	2.638804	-1.594878	-0.059990
12	6	0	3.007485	-2.097717	-1.461164
13	1	0	3.518853	-3.060147	-1.375461
14	1	0	2.122229	-2.225128	-2.081714
15	1	0	3.682494	-1.390812	-1.950283
16	6	0	1.645964	-2.546215	0.621245
17	1	0	2.085272	-3.543101	0.710299
18	1	0	1.399177	-2.188551	1.624952
19	1	0	0.725138	-2.626264	0.042833
20	6	0	3.922318	-1.535170	0.775519
21	1	0	4.304120	-2.551949	0.885151
22	1	0	4.699875	-0.950271	0.279136
23	1	0	3.752193	-1.149705	1.784450
24	7	0	2.016931	-0.246991	-0.165500
25	8	0	3.591098	1.467576	0.479084
26	1	0	4.268269	0.991471	0.962947
27	1	0	2.283861	0.537860	1.769927
28	6	0	-0.711658	1.991926	-1.088967
29	3	0	-1.449926	0.281543	-2.205991
30	6	0	-3.675744	0.607722	-0.249876
31	8	0	-4.251023	1.621860	-0.197983
32	7	0	-3.067878	-0.417728	-0.420131

33	6	0	-2.782177	-1.575713	0.457717
34	6	0	-2.254502	-2.693296	-0.438541
35	1	0	-3.013258	-2.992020	-1.166221
36	1	0	-1.361012	-2.364425	-0.973016
37	1	0	-1.997929	-3.564306	0.169343
38	6	0	-4.072446	-2.008197	1.155685
39	1	0	-3.876893	-2.882625	1.781176
40	1	0	-4.458627	-1.210619	1.796541
41	1	0	-4.838767	-2.272311	0.423112
42	6	0	-1.726766	-1.151784	1.481105
43	1	0	-2.106683	-0.352139	2.122077
44	1	0	-1.459725	-2.004508	2.110459
45	1	0	-0.824996	-0.795434	0.981901

TS

Center Number	Atomic Number	Atomic Type	Coordinates (Angstroms) X	Y	Z
1	6	0	-0.662287	1.049414	0.257571
2	6	0	-1.775545	1.761011	-0.159182
3	6	0	-1.671851	3.128977	-0.284023
4	6	0	0.550424	2.996120	0.326286
5	6	0	-1.069200	-0.371426	0.374375
6	1	0	-2.508834	3.753695	-0.582096
7	1	0	1.461829	3.576651	0.464085
8	7	0	-0.506926	3.742136	-0.030888
9	8	0	-0.323308	-1.283729	0.747737
10	6	0	-2.938578	0.831598	-0.363637
11	6	0	-3.059168	-1.786773	-0.172151
12	6	0	-3.075089	-2.537619	1.165070
13	1	0	-3.621990	-3.476946	1.048061
14	1	0	-2.064131	-2.762044	1.502513
15	1	0	-3.577992	-1.937925	1.928007
16	6	0	-2.316688	-2.590237	-1.248829
17	1	0	-2.803566	-3.558744	-1.388430
18	1	0	-2.335046	-2.055333	-2.202476
19	1	0	-1.279223	-2.764029	-0.962539
20	6	0	-4.507572	-1.580381	-0.627773
21	1	0	-4.935993	-2.560129	-0.847073
22	1	0	-5.121743	-1.131497	0.155599
23	1	0	-4.579309	-0.986836	-1.542650
24	7	0	-2.370063	-0.479319	0.007561
25	8	0	-4.009056	1.229692	0.455636
26	1	0	-4.833206	0.920872	0.075402
27	1	0	-3.254084	0.803614	-1.411250
28	6	0	0.592414	1.599104	0.509528
29	3	0	1.333889	-0.412081	1.466266
30	6	0	2.879456	0.955876	-0.128090

31	8	0	3.153432	1.968939	-0.649868
32	7	0	2.954044	-0.203727	0.297607
33	6	0	4.120361	-1.069540	-0.055820
34	6	0	3.945942	-2.350693	0.755652
35	1	0	3.967350	-2.133252	1.827047
36	1	0	2.993176	-2.829641	0.514633
37	1	0	4.752457	-3.053695	0.532559
38	6	0	5.430869	-0.375257	0.319594
39	1	0	6.275799	-1.035630	0.107448
40	1	0	5.570521	0.545701	-0.252630
41	1	0	5.445004	-0.128733	1.384348
42	6	0	4.077793	-1.378131	-1.553791
43	1	0	4.188275	-0.465701	-2.146255
44	1	0	4.893080	-2.056339	-1.819789
45	1	0	3.130334	-1.852911	-1.820001

----

[3] X-ray Diffraction Analysis

Table S1. Crystal structure, data collection and refinement parameters.

**Compound**	**7**	**9b**	**9c**	**9e**	**10**	**11**	**12**
**Crystal data**							
CCDC	2548707	2548713	2548717	2548718	2548719	2548724	2548728
Chemical formula	$C_{11}H_{14}N_3O_3 \cdot 0.66\ CH_3OH$	$C_{14}H_{22}N_2O_2Si$	$C_{18}H_{19}N_3O_3$	$C_{16}H_{23}N_3O_2S$	$2(C_{21}H_{32}N_4O_4)\ CH_3OH$	$C_{18}H_{17}N_3O_3$	$C_{16}H_{21}N_3O_3$
Formula weight	227.39	278.42	325.36	321.43	841.05	323.34	303.36
Crystallization conditions	DCE : CH_3OH (1:1 v/v)	DCM : CH_3OH (1:1 v/v)	DCM : CH_3OH (1:1 v/v)	DCM	DCM : CH_3OH (1:1 v/v)	DCM : AcOEt (7:3 v/v)	DCM
Crystal system	trigonal	monoclinic	triclinic	triclinic	monoclinic	triclinic	orthorombic
Space group	$P3_1$	$P2_1/n$	P-1	P-1	Pc	P-1	$Pca2_1$
Temperature (K)	100.00(10)	100.00(10)	100.00(10)	99.99(10)	99.98(10)	99.99(10)	100.00(10)
a [Å]	12.7280(2)	12.71205(9)	7.3343(2)	11.46814(19)	16.0841(3)	7.0677(3)	11.55733(6)
b [Å]	12.7280(2)	15.66989(12)	10.4512(2)	11.5139(2)	9.69401(14)	10.4728(5)	8.91359(12)
c [Å]	6.2870(2)	16.08590(11)	11.7076(3)	12.7865(3)	16.6421(3)	11.4927(4)	29.8705(4)
α [°]	90	90	114.704(2)	90.8621(16)	90	72.879(4)	90
β [°]	90	91.6760(6)	98.297(2)	95.1941(16)	115.177(3)	84.100(3)	90
γ [°]	120	90	97.880(2)	93.8363(14)	90	71.736(4)	90
V [Å^3]	882.05(4)	3202.88(4)	787.06(4)	1677.29(6)	2348.31(9)	771.97(6)	3077.18(8)
**Z**	3	8	2	4	2	2	8
**Z’**	1	4	1	2	1	1	2
d_{calc} [g/cm^3]	1.284	1.155	1.373	1.273	1.189	1.391	1.310
Crystal dimensions [mm]	0.7 × 0.05 × 0.04	0.6 × 0.2 × 0.1	0.5 × 0.1 × 0.02	0.05 × 0.01 × 0.01	0.3 × 0.15 × 0.02	0.4 × 0.05 × 0.04	0.3 × 0.1 × 0.04
Radiation type	CuKα	CuKα	CuKα	CuKα	CuKα	CuKα	CuKα
μ [mm^{-1}]	0.753	1.298	0.778	1.801	0.684	0.793	0.750
**Data collection**							
Reflections measured	7437	45348	19708	50045	25679	19355	17958

Range/indices (*h*, *k*, *l*)	-14, 15<br>-16, 15<br>-7, 7	-15, 16<br>-19, 19<br>-19, 20	-9, 8<br>-12, 13<br>-14, 14	-14, 13<br>-14, 14<br>-16, 15	-19, 20<br>-11, 12<br>-20, 19	-8, 8<br>-13, 12<br>-13, 14	-10, 14<br>-11, 10<br>-35, 36
θ (max, min) [°]	76.499, 4.010	77.048, 3.939	77.251, 4.256	77.205, 3.472	76.878, 3.036	76.704, 4.025	77.048, 2.959
Total no. of unique data	2195	6621	3060	6660	8122	3030	5679
No. of observed data, I > 2σ(I)	2128	6056	2773	5874	7364	2700	5050
R_{int}	0.039	0.046	0.048	0.049	0.051	0.047	0.047
**Refinement**							
$R [F^2 > 2\sigma (F^2)]$	0.028	0.036	0.039	0.050	0.040	0.038	0.041
$wR(F^2)$	0.068	0.099	0.104	0.135	0.108	0.103	0.115
S	1.081	1.052	1.062	1.100	1.080	1.052	1.061
No. of reflections	2195	6621	3060	6660	8122	3030	5679
No. of parameters	140	357	225	451	576	224	413
No. of restraints	1	0	0	0	2	0	1
H-atom treatment	H atoms parameters constrained	H atoms parameters constrained	H atoms treated by a mixture of independent and constrained refinement	H atoms treated by a mixture of independent and constrained refinement	H atoms treated by a mixture of independent and constrained refinement	H atoms treated by a mixture of independent and constrained refinement	H atoms treated by a mixture of independent and constrained refinement
Δρ (min, max), e/Å^3	-0.15/0.10	-0.37/0.32	-0.21/0.28	-0.44/0.52	-0.23, 0.26	-0.21/0.24	-0.24/0.30
Absolute structure parameter	0.26(13)	-	-	-	0.08(11)	-	0.3(3)

Structural analysis

X-ray diffraction analysis was performed to confirm the structures of the synthesized compounds: **7**, **9b**, **9c**, **9e**, **10**, **11**, and **12**. All single crystals were obtained by re-crystallization from corresponded solvents of their mixtures. All crystallographic data are listed in **Table S1.** Crystal structures of listed compounds are reported for the first time. The analyzed molecules crystallize in various space groups, indicating that used modifications have significant effects on their crystal packing.

Compound 9b

Compound **9b** crystallizes in the monoclinic $P2_1/n$ space group with two molecules per asymmetric unit. The crystal structure confirms that the crystallized molecule is the R-enantiomer at the C7 carbon atom (**Figure S21a**). Analysis of the molecular packing shows that the molecules are organized into four-molecule blocks stabilized by 1.991 Å O22–H22···N1 hydrogen bonds, which are arranged along the crystallographic *b*-axis into infinite columns (**Figure S21c**). The molecular cores are located within the columns, while the hydrophobic *tert*-butyl and trimethylsilyl groups are exposed on the outside. Hydrophobic interactions stabilize adjacent columns (**Figure S21b**).

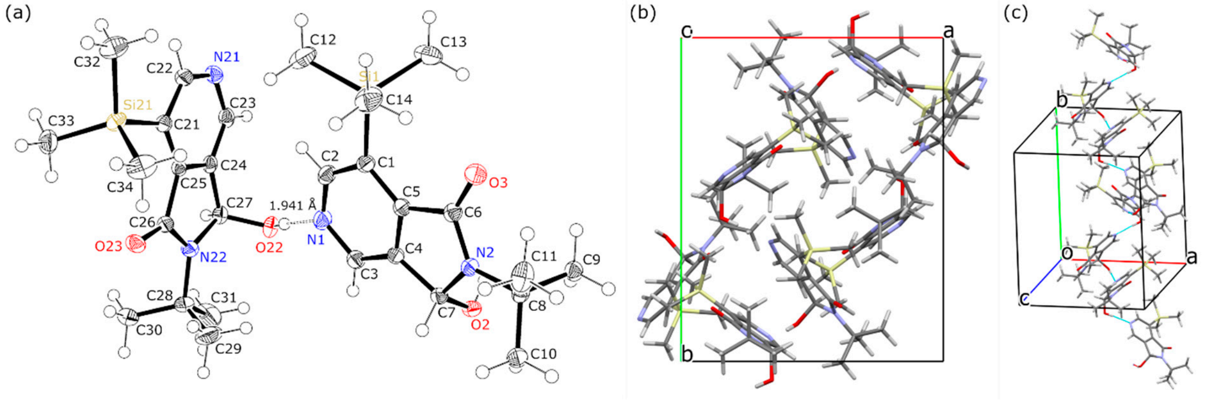

Figure S21. (a) Thermal ellipsoid representation of the asymmetric unit for the monoclinic ($P2_1/n$, Z' = 2) form of compound **9b**. The anisotropic displacement parameters (ADPs) are shown at the 50% probability level. (b) View of molecular packing along the crystallographic *c*-axis. (c) Perspective view of molecular packing with indications of intermolecular hydrogen bonds.

Compound 9e

Compound **9e** crystallizes in the triclinic *P*-1 space group with two molecules per asymmetric unit. Experimental results confirm that the crystal contains unequal volumes of the R- and S-enantiomers, as observed at the C7/C27 carbon atom. One molecule is the pure S-enantiomer, while the other has an R to S ratio of 86.9 to 13.1%, based on occupancy refinement. The molecular conformation is stabilized by an intramolecular N3–H3···O3 (N23–H23···O23) hydrogen bond measuring 1.789 (2.023) Å. An intermolecular hydrogen bond, O22B–H22B···O2, is also present, measuring 1.800 Å (**Figure S22a**). Analysis of the crystal packing reveals additional intermolecular hydrogen bonds between the molecules: O2–H2···N21(i) at 1.967 Å and O22A–H22A···N2(ii) at 1.944 Å (symmetry codes: (i) 1–x, 1–y, 1–z; (ii) –x, 1–y, 1–z). The molecular rings are arranged in planes oriented diagonally in the b × c plane, perpendicular to this plane, and their positions are further stabilized by π-π stacking interactions (**Figure S22b**, **c**).

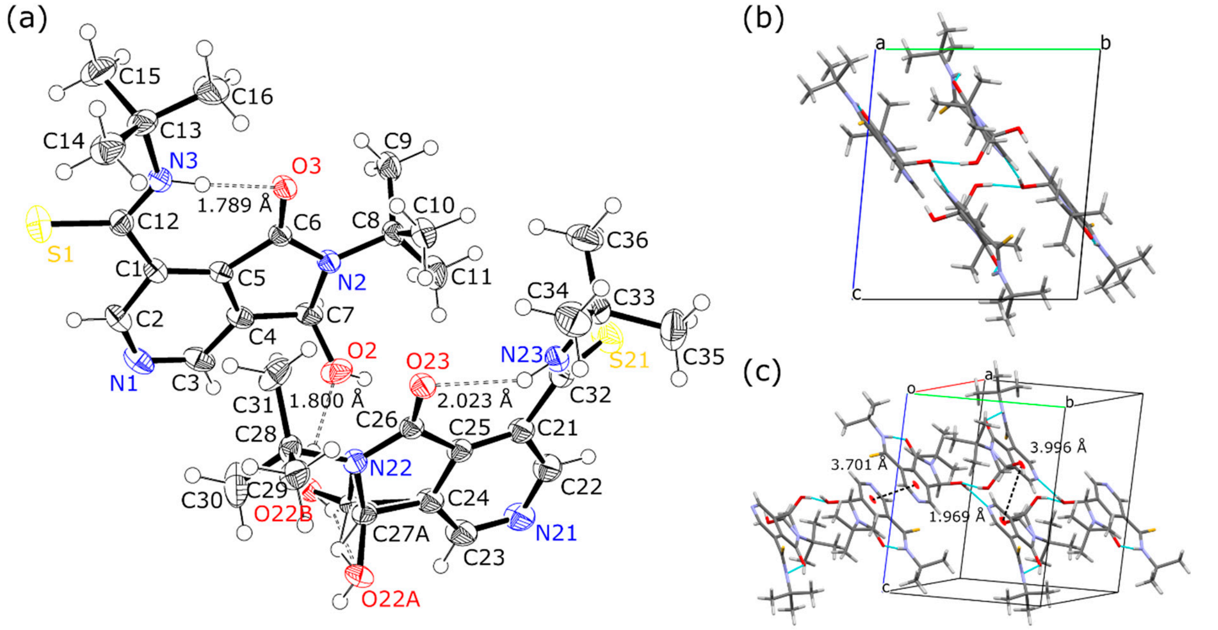

Figure S22. (a) Thermal ellipsoid representation of the asymmetric unit for the triclinic (*P*-1, Z' = 2) form of compound **9e**. The anisotropic displacement parameters (ADPs) are shown at the 50% probability level. (b) View of molecular packing along the crystallographic *a*-axis shows the layered organization of molecules. (c) Perspective view of molecular packing with indications of intermolecular hydrogen bonds and π–π stacking interactions.

Compound 10

Compound **10** crystallizes in the monoclinic $P2_1/c$ space group with two molecules per asymmetric unit. Experimental results confirm that the crystal consists of equal volumes of the R- and S-enantiomers, observed at the C7/C37 carbon atom. The molecular conformation is stabilized by an intramolecular N3–H3···O3 (N33–H33···O33) hydrogen bond measuring 1.899 (1.840) Å (**Figure S23a**). Analysis of the crystal packing shows that the molecules are organized into four-molecule blocks with alternating R- and S-molecules, stabilized by intermolecular N34–H34···O1 hydrogen bonds measuring 2.270 Å, which are arranged along the crystallographic *a*-axis into infinite columns (**Figure S23c**). The molecular cores are located within the columns, while the hydrophobic *tert*-butyl residues are exposed on the sides along the *c*-axis direction, and hydrophobic interactions stabilize adjacent blocks (**Figure S23b**).

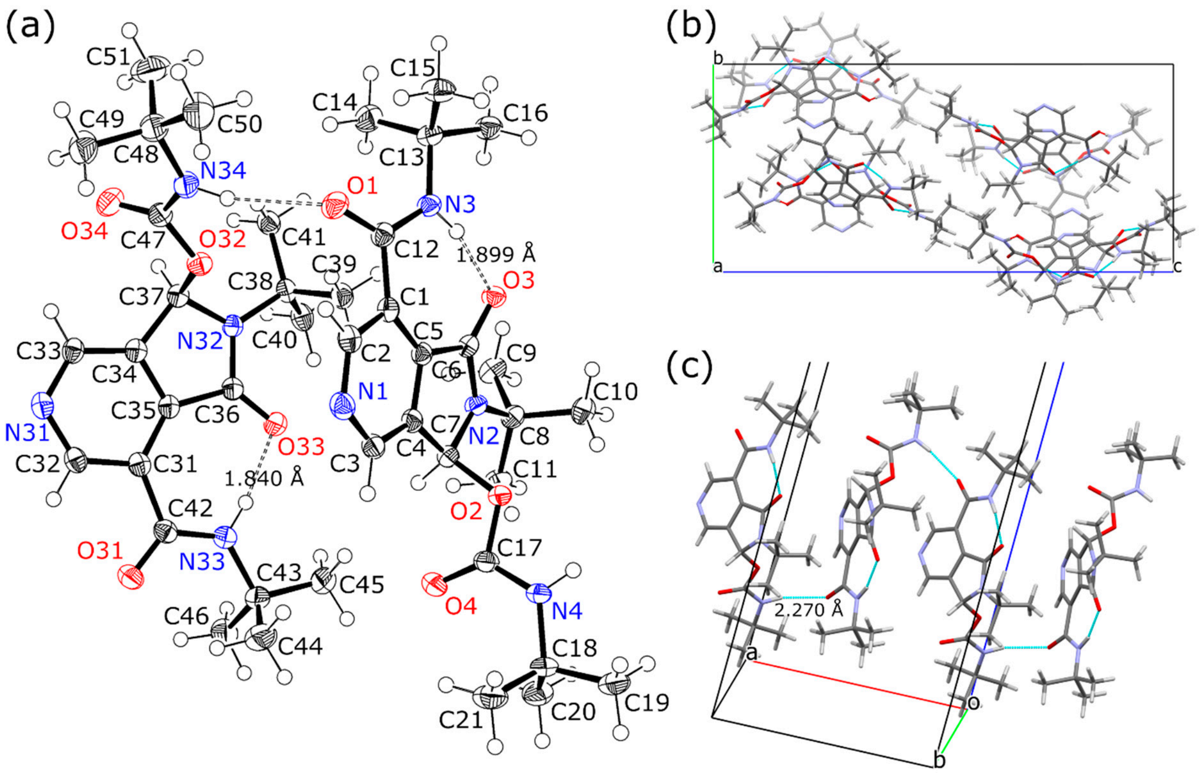

Figure S23. (a) Thermal ellipsoid representation of the asymmetric unit for the monoclinic ($P2_1/c$, Z' = 2) form of compound **10**. The anisotropic displacement parameters (ADPs) are shown at the 50% probability level. (b) View of molecular packing along the crystallographic *a*-axis shows hydrophobic interactions between side chains. (c) Perspective view of molecular packing with indication of intermolecular hydrogen bonds.

Compound 11

Compound **11** crystallizes in the triclinic *P*-1 space group with one molecule per asymmetric unit. The molecular conformation is stabilized by an intramolecular N3–H3···O3 hydrogen bond measuring 1.854 Å (**Figure S24a**). Analysis of the crystal packing shows that the molecules form stacked columns parallel to the crystal *a*-axis, consisting of alternating molecules stabilized by π–π stacking interactions between phenyl and pyridine rings, with distances ranging from 3.599 to 3.647 Å (**Figure S24c**). Adjacent columns interact through hydrophobic interactions involving *t*-butyl groups (**Figure S24b**). No solvent is present in the crystal structure.

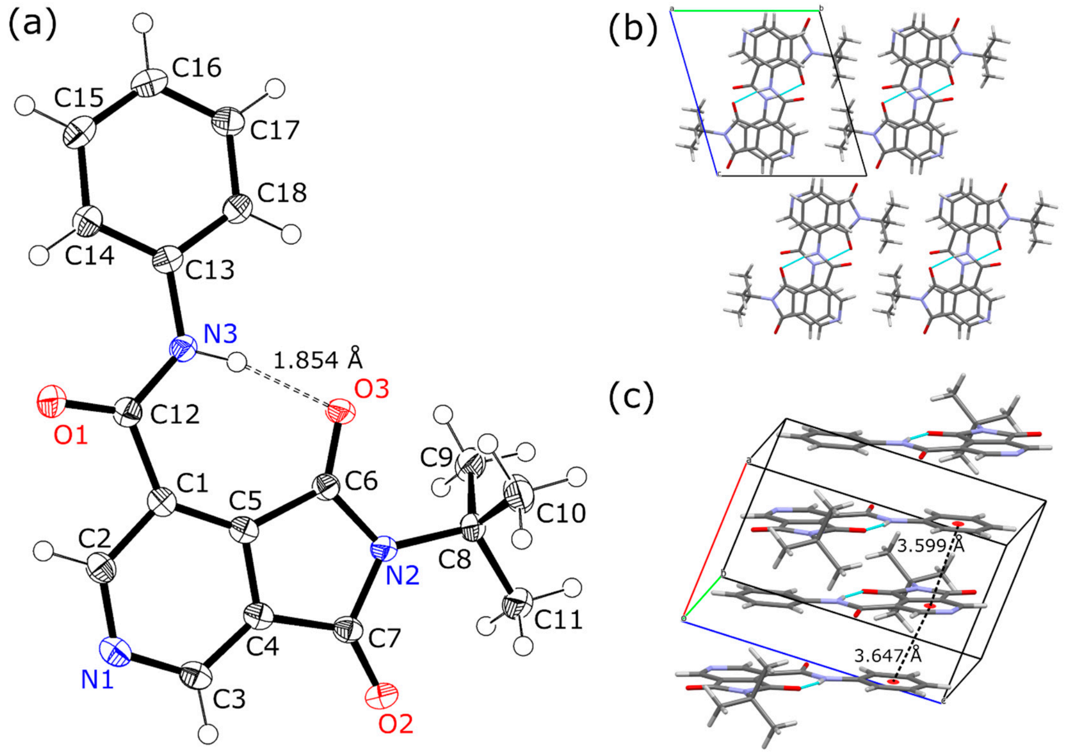

Figure S24. (a) Thermal ellipsoid representation of the asymmetric unit for the triclinic (*P*-1, Z' = 1) form of compound **11**. The anisotropic displacement parameters (ADPs) are shown at the 50% probability level. (b) View of molecular packing along the crystallographic a-axis shows hydrophobic interactions between side chains. (c) Perspective view of molecular packing indication π–π stacking interactions.

Compound 12

Compound **12** crystallizes in the orthorhombic *Pca*2₁ space group with two molecules per asymmetric unit. The conformation of each molecule is stabilized by an intramolecular N3–H3···O3 hydrogen bond measuring 1.900–1.902 Å (**Figure S25a**). Analysis of the crystal packing shows that the molecules are arranged in alternating layers, maintaining a herringbone-like pattern when viewed along the *a*-axis. In the b × c plane, the molecules are arranged alternately, creating hydrophobic regions composed of *tert*-butyl residues and hydrophilic regions with exposed rings (**Figure S25b, c**)

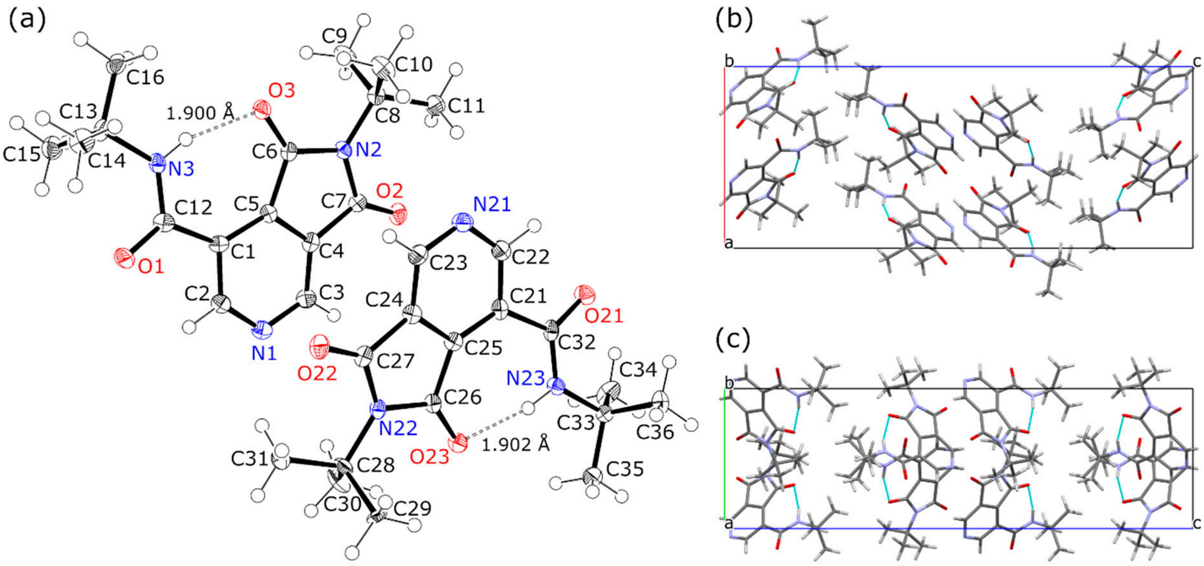

Figure S25. (*a*) Thermal ellipsoid representation of the asymmetric unit for the orthorombic (*Pca*2₁, Z' = 2) form of compound **12**. The anisotropic displacement parameters (ADPs) are shown at the 50% probability level. (*b*) View of molecular packing along the crystallographic *b*-axis. (*c*) View of molecular packing along the crystallographic *a*-axis.
